# Effects of LC *n*-3 PUFA Supplementation on Muscle Pain, Function, and Damage Markers in Healthy Young to Middle-Aged Adults Following Acute or Chronic Exercise: A Systematic Review and Meta-Analysis of Randomized Controlled Trials

**DOI:** 10.3390/nu18091447

**Published:** 2026-04-30

**Authors:** Elham Yaghoobi, Fereshteh Pashaei, Giselle L. Allsopp, Matthew Retallack, Nicholas Charalambous, Rhiannon M. J. Snipe, Christopher S. Shaw, Greg M. Kowalski, Clinton R. Bruce, Angus M. Hunter, Martin C. Refalo, Gunveen Kaur, Gavin Abbott, D. Lee Hamilton

**Affiliations:** 1 Institute for Physical Activity and Nutrition, Faculty of Health, School of Exercise and Nutrition Sciences, Deakin University, Waurn Ponds, Geelong, VIC 3216, Australia; e.yaghoobi@deakin.edu.au (E.Y.); g.allsopp@deakin.edu.au (G.L.A.); r.snipe@deakin.edu.au (R.M.J.S.); chris.shaw@deakin.edu.au (C.S.S.); clinton.bruce@deakin.edu.au (C.R.B.); martin.refalo@deakin.edu.au (M.C.R.); gunveen.kaur@deakin.edu.au (G.K.); gavin.abbott@deakin.edu.au (G.A.); 2 Department of Clinical Nutrition and Dietetics, Faculty of Nutrition Sciences and Food Technology, National Nutrition and Food Research Institute, Shahid Beheshti University of Medical Sciences, Tehran 19816-19573, Iran; fereshteh.pashai@gmail.com; 3 School of Medicine, Faculty of Health, Deakin University, Waurn Ponds, Geelong, VIC 3220, Australia; matthew.retallack@deakin.edu.au (M.R.); nic_chara@hotmail.com (N.C.); 4 Institute for Physical Activity and Nutrition, Metabolic Research Unit, School of Medicine, Faculty of Health, Deakin University, Waurn Ponds, Geelong, VIC 3216, Australia; greg.kowalski@deakin.edu.au; 5 Department of Sport Science, Nottingham Trent University, Nottingham NG11 8NS, UK; angus.hunter@ntu.ac.uk; 6 Faculty of Health Sciences and Sport, University of Stirling, Stirling FK9 4LA, UK; 7 School of Medicine, University of Aberdeen, Aberdeen AB25 2ZD, UK; 8 Health Through Physical Activity, Lifestyle and Sport Research Centre, University of Cape Town, Cape Town 7700, South Africa

**Keywords:** long-chain omega-3 polyunsaturated fatty acids, eicosapentaenoic acid, docosahexaenoic acid, exercise-induced muscle damage, recovery, muscle soreness, muscle function

## Abstract

***Background:*** Supplementation with long-chain omega-3 polyunsaturated fatty acids (LC *n*-3 PUFAs), particularly eicosapentaenoic acid (EPA) and docosahexaenoic acid (DHA), may mitigate exercise-induced muscle damage (EIMD) and enhance post-exercise recovery. However, the systematic reviews/meta-analyses evaluating these effects across populations and exercise models are limited and do not provide dosing recommendations. ***Objective:*** This systematic review and meta-analysis aimed to evaluate the effects of LC *n*-3 PUFA supplementation on key post-exercise recovery outcomes, including muscle soreness, muscle function, and muscle damage biomarkers in healthy adults. ***Methods:*** Following the PRISMA guidelines, a comprehensive search of PubMed, Scopus, and clinical trial registry databases was conducted (to January 2025). All studies that met the inclusion criteria underwent appropriate methodological quality assessments using established tools. The data were extracted for inputting into random-effects models, with effect sizes reported as Hedges’ g and 95% confidence intervals (CIs). Heterogeneity was assessed using the *I*^2^ statistic. ***Results:*** Among the 2539 records, 43 studies met the inclusion criteria for the systematic review, and nine met the inclusion criteria for the meta-analysis. The effect of LC *n*-3 PUFA supplementation on recovery outcomes was equivocal, with significant methodological limitations noted across the literature. However, the meta-analysis of nine placebo-controlled, eccentric exercise trials demonstrated that LC *n*-3 PUFA supplementation significantly reduced delayed onset muscle soreness (DOMS) (Hedges’ g = −0.75; 95% CI: −1.14 to −0.36), creatine kinase (CK) (Hedges’ g = −0.40; 95% CI: −0.70 to −0.10), and muscle swelling (Hedges’ g = −0.45; 95% CI: −0.83 to −0.07), and significantly improved muscle strength (Hedges’ g = 0.45; 95% CI: 0.07 to 0.83) and range of motion (ROM) (Hedges’ g = 0.93; 95% CI: 0.33 to 1.53) at peak impairment compared with placebo. ***Conclusions:*** LC *n*-3 PUFA supplementation may support recovery from EIMD. However, due to the methodological limitations across the literature base it was not possible to assess effective dosing strategies. Future studies should address dose–response and duration requirements and incorporate objective assessments of omega-3 status (e.g., the Omega-3 Index [O3I] or comparable biomarkers) alongside standardized compliance measures. These approaches are necessary to determine effective dosing strategies and to test the relationship between omega-3 status and recovery outcomes.

## 1. Introduction

Exercise-induced muscle damage (EIMD) is a widely recognized consequence of eccentric exercise, manifesting as impaired muscle function, delayed onset muscle soreness (DOMS), and extended recovery periods, all of which can negatively impact athletic performance [1]. DOMS typically occurs between 12 and 48 h post-exercise, peaks within 24 to 72 h, and may persist for up to seven days, limiting both athletes and beginners from returning to physical activity [1]. This prolonged recovery period not only increases the risk of further injury but also disrupts training consistency and progress [2,3,4]. The physiological responses to EIMD are complex and involve a series of interconnected events. These include the disruption of sarcomeres and muscle plasma membranes leading to the release of muscle-specific enzymes, and the activation of inflammatory and oxidative stress pathways causing tissue swelling and reductions in muscle strength and range of motion [5]. While inflammation is critical for muscle repair and adaptation, excessive or sustained inflammation may exacerbate muscle damage, potentially delaying recovery and impairing overall performance [5,6].

Common management strategies for EIMD typically include reducing exercise intensity or resting the affected muscles, yet these approaches can result in significant training interruptions and decreased motivation, particularly among beginners [7]. Other strategies target the inflammation pathways with non-steroidal anti-inflammatory drugs (NSAIDs), which help with pain relief. However, prolonged NSAID use may lead to adverse side effects and inhibit long-term training adaptations [7,8,9,10]. Alternative recovery methods, such as low-intensity exercise and massage, have demonstrated some effectiveness for reducing pain, but do not fully restore muscle function and performance [7].

Given the proposed role of inflammation and oxidative stress in the pathogenesis of EIMD, antioxidant, polyphenol (e.g., resveratrol, quercetin), and polyunsaturated fatty acid (PUFA) supplementation have gained considerable attention as potential interventions to reduce muscle damage and enhance recovery [11,12,13]. PUFAs are dietary fats characterized by two or more double bonds and are found in abundance in fish, seeds, nuts, and certain vegetable oils [14]. They are recognized for their anti-inflammatory, cardioprotective, and metabolic benefits [14]. Among these, long-chain omega-3 PUFAs (LC *n*-3 PUFAs), particularly eicosapentaenoic acid (EPA; 20:5*n*-3) and docosahexaenoic acid (DHA; 22:6*n*-3) derived from fish oil (or algae), have demonstrated promising anti-inflammatory and analgesic properties [15,16,17]. Incorporation of EPA and DHA into skeletal muscle membranes may alter membrane fluidity and structural integrity, potentially protecting tissue integrity [18]. Additionally, LC *n*-3 PUFA supplementation has been shown in some studies to reduce muscle soreness, damage markers and inflammatory markers, suggesting a possible role in alleviating DOMS and supporting recovery [18,19]. Previous systematic reviews and meta-analyses [20,21,22,23] have predominantly focused on isolated outcomes, such as DOMS, biomarkers of damage, strength preservation or selected inflammatory markers, often within narrower supplementation protocols and specific populations. However, the considerable heterogeneity of the methodologies across LC *n*-3 PUFA supplementation studies has contributed to substantial variability in the reported outcomes, and discerning the minimum effective dose/duration protocols appears impossible from current reviews. Therefore, this systematic review and meta-analysis aimed to comprehensively evaluate the efficacy of LC *n*-3 PUFA supplementation for attenuating the adverse effects of EIMD and promoting recovery in both trained and untrained participants across acute and chronic exercise protocols, and to determine whether evidence-based dosing recommendations could be established.

## 2. Methods

### 2.1. Study Overview Component

The primary objective of this review was to assess the efficacy of LC *n*-3 PUFA supplementation trials for promoting recovery from EIMD. The aim was to determine whether LC *n*-3 PUFAs can attenuate DOMS, improve muscle function (strength, ROM), reduce circulating blood-based markers of muscle damage (creatine kinase (CK), lactate dehydrogenase (LDH), and myoglobin (Mb)), inflammation, oxidative stress, and muscle swelling following muscle damage or intensive exercise protocols.

### 2.2. Literature Search and Study Selection

A systematic search of the literature was conducted across multiple databases, including PubMed, Scopus, and clinical trial registries, to identify studies published up to January 2025. The search strategy used various combinations of the following search terms: “muscle damage” OR “exercise induced muscle damage” OR “creatine kinase” OR “CK” OR “Lactate Dehydrogenase” OR “LDH” OR “exercise recovery” OR “eccentric exercise” OR “EIMD” OR “delayed onset muscle soreness” OR “DOMS” OR “muscle soreness” OR “muscle function” OR “muscle strength” AND “Omega-3” OR “omega3” OR “n3” OR “*n*-3” OR “fish oil” OR “krill oil” OR “algae oil” OR “mussel oil” OR “seafood” OR “EPA” OR “DHA” OR “DPA” NOT “older” OR “elderly” OR “sarcopenia” OR “cancer” OR “dialysis”. Whenever possible, the database results were restricted to include only studies involving human participants.

The screening process commenced with the removal of duplicates and the exclusion of ineligible studies at each stage using Covidence (www.covidence.org). Subsequently, the titles and abstracts of the studies were screened to eliminate irrelevant or off-topic research. The full texts of the remaining studies were then retrieved and evaluated according to the established inclusion and exclusion criteria. Two blinded authors independently reviewed each article, resolving any disagreements through discussion. If a consensus could not be reached, a third author was consulted.

### 2.3. Inclusion and Exclusion Criteria

**Figure 1:**
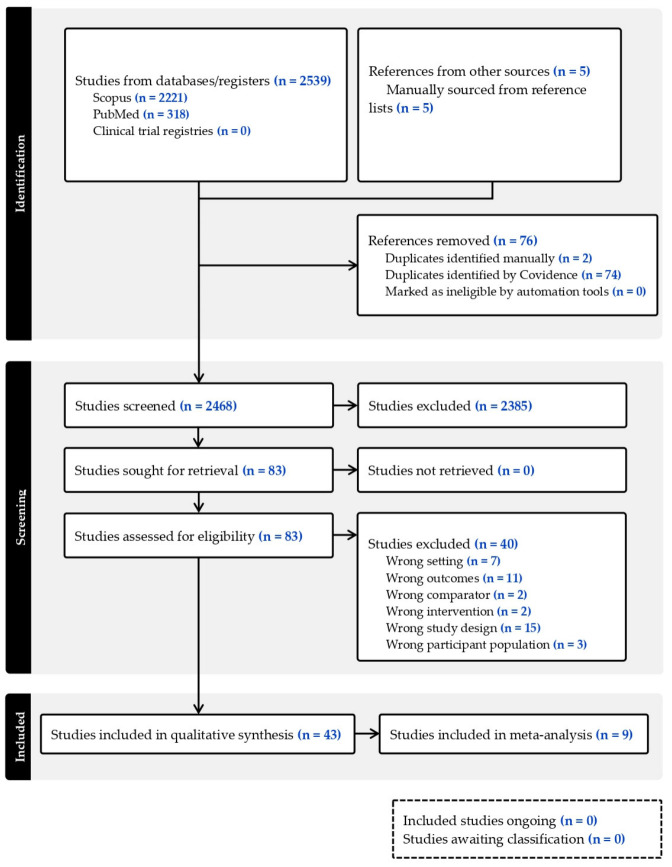
PRISMA flow diagram showing the study selection process.

The inclusion criteria were as follows: (a) exercise interventions combined with any of the following supplements: LC *n*-3 PUFAs, fish oil, krill oil, mussel oil, algae oil, or seafood; (b) healthy young to middle-aged adults (18–40 years old); (c) a placebo-controlled group undergoing the same exercise intervention, with the outcomes experimentally compared through pre- to post-intervention changes in pain and/or muscle function and/or markers of muscle damage and/or oxidative stress; and (d) placebo-controlled studies (not limited to randomized controlled trials (RCTs)), as well as reviews, meta-analyses, and systematic reviews. While the reviews, meta-analyses, and systematic reviews did not have their data extracted, they were used to screen reference lists for any studies that may have been missed during our search. Studies were excluded if they met any of the following criteria: (a) involved a population with any chronic disease; (b) did not include an exercise intervention; (c) failed to assess muscle soreness, and/or functional parameters, and/or relevant blood markers of muscle damage related to a muscle-damaging or intensive exercise protocol; or (d) were classified as conference papers, book chapters, or conference proceedings. An overview of the study identification, screening, eligibility assessment, and inclusion process is presented in the PRISMA flow diagram in Figure 1.

### 2.4. Assessment of Study Quality

The quality and internal validity of the included studies were assessed using the Cochrane risk of bias tool (RoB), which evaluates the methodological rigor of randomized controlled trials (RCTs) through a domain-based evaluation [24] Each study was classified as having either low risk, high risk, or unclear risk for each of the seven items (see Supplementary Figures S1 and S2). Two authors independently judged the risk of bias, resolving any disagreements through discussion. If consensus could not be reached, a third author was consulted to ensure consistency and transparency throughout the evaluation process.

The McMaster Quality Assessment Tool developed by McMaster University Occupational Therapy Evidence-Based Practice Research Group was used to evaluate the methodological quality of the studies included in this review. Although it was originally developed for assessing qualitative research [20,25], it remains a thorough tool for assessing research quality as it assesses 16 key criteria comprising quality domains also relevant to quantitative research. The McMaster tool applies a score of 1 if the criterion is met and 0 if it is not, with a maximum possible score of 16 (see Supplementary Figure S3). In addition, the PEDro scale was used to assess the methodological quality of randomized controlled trials (RCTs) included in this review. The PEDro scale consists of 11 items designed for rehabilitation-style clinical trials, with each criterion (except for eligibility) scored as 1 if met or 0 if not (see Supplementary Figure S4) [26].

### 2.5. Data Extraction for Systematic Review

The key information was extracted from the papers and charted in a table format (Table 1). The participant characteristics included: (a) training status (e.g., sedentary, recreationally active, trained/developmental, highly trained/national level, elite/international level, or world class), categorized by the caliber classification in McKay et al. (2022) [27] and, where possible, their sport or training background was included; (b) sex (including if menstrual cycle status of females was controlled or monitored); and (c) age. The study characteristics recorded were: (a) publication date, (b) first author, (c) sample size, (d) intervention groups and protocol details, (e) supplementation specifics, (f) intervention duration, (g) compliance method, (h) muscle damage stimulus, (i) outcome measures, (j) reported pain levels, (k) omega-3 index (O3I) or LC *n*-3 PUFA status changes, and (l) differences between placebo and intervention groups for all relevant outcomes.

### 2.6. Data Extraction for Meta-Analysis

For the quantitative synthesis for the meta-analysis, the relevant variables were extracted from studies that met the following criteria: (1) placebo-controlled, eccentric exercise trials; (2) clearly specified treatment group brand (i.e., the specific commercial product and/or manufacturer used) and placebo treatment, in addition to stating the dosing strategy; (3) assessed muscle pain using the Visual Analog Scale (VAS) and/or muscle function outcomes (1RM or MVIC or equivalent or ROM) and/or CK, and/or muscle swelling (UAC, muscle thickness); and (4) demonstrated a significant positive change in the LC *n*-3 PUFA status in the treatment group. The numerical outcomes were extracted from the statistically significant time point or, when not reported or where there were multiple statistically significant time points, from the time point corresponding to peak dysfunction in the placebo group. Peak dysfunction was defined as the time point at which muscle pain was highest, muscle strength and ROM were most impaired, and swelling and CK concentrations were most elevated. The data were extracted as the mean and standard deviation or standard error (with standard errors converted to SD) for the control and intervention groups. When outcome data were not presented in tables or text and the corresponding authors could not be reached, the data were extracted using WebPlotDigitizer (Web Plot Digitizer, V.3.11. Austin, TX, USA) [71]. Where the data were graphed as the % or fold change from baseline, resulting in a baseline reading of 0 or 100 with no discernable error bars, we extracted the post-damage data only for calculating the treatment vs control effect sizes. The meta-analysis results were presented using forest plots.

### 2.7. Data Synthesis

The meta-analyses were conducted in Stata version 18, with separate models for the DOMS, muscle strength, ROM, CK, and swelling outcomes. Hedges’ g effect sizes were calculated for each study and the pooled effects were estimated using restricted maximum likelihood random-effects models, with multilevel models used for the DOMS and strength outcomes due to one study (Mackay et al., 2023) [66] providing outcome data for both hamstring and quad muscles, which were used as separate effects in the models. In one study [68], the data from the three intervention groups were combined into a single intervention group following the Cochrane Handbook formula for combining groups [72]. The effect sizes (Hedges’ g) were interpreted as small (0.2), moderate (0.5), and large (0.8) [73]. Heterogeneity was assessed using *I*^2^, with values of 25%, 50%, and 75% representing low, moderate, and high heterogeneity, respectively [74]. Potential publication bias was assessed via inspection of funnel plots, while leave-one-out analyses were conducted to assess whether the findings were sensitive to exclusion of any individual studies.

## 3. Results

### 3.1. Studies Selected

A total of 2539 publications were identified through the database search, with an additional five publications found by reviewing reference lists of identified studies and relevant review articles (Figure 1). After removing duplicates, 2385 publications were excluded based on title and abstract screening. The remaining 83 publications underwent full-text reviews, resulting in the exclusion of an additional 40 studies. Figure 1 outlines the selection process and reasons for exclusion. Forty-three publications met the inclusion criteria (Table 1), of which nine met the inclusion criteria for the meta-analysis.

### 3.2. Risk of Bias Assessment

Of the 43 studies in this review, 38 were RCTs. Internal validity of RCTs was assessed using the Cochrane Collaboration’s risk of bias tool (see Supplementary Figures S1 and S2). All but four RCTs reported a randomization process. Only 11 (28.9%) provided details about the randomization component used in sequence generation. In most studies (31 out of 38), allocation concealment was deemed high risk. Thirty-one studies clearly described participant and personnel blinding. However, only two (5.2%) gave sufficient details on the blinding for the outcome assessment. Seventeen studies (44.7%) had a high risk of bias due to incomplete outcome data. In most cases (41 out of 43), the selective reporting bias was low. Nine studies (23.6%) were judged to be at high risk of bias due to deviations from the intended interventions.

Following the Cochrane assessment, we also applied the McMaster Quality Assessment Tool to all included studies [25]. Of the 43 studies, only four achieved the maximum score of 16 [27,41,66,68]. Rajabi et al. [39] received the lowest score of 10, while the others scored between 11 and 15, as shown in Supplementary Figure S3.

Additionally, this review also used the PEDro Scale [26]. Loss et al. [60] achieved the highest PEDro rating, while Buonocore et al. [55] earned the lowest. Supplementary Figure S4 illustrates that the remaining studies scored between 4 and 9.

As a quality control step for the meta-analysis component of this review, the funnel plots of each meta-analysis were inspected, and they showed no evidence of publication bias (Supplementary Figure S5). Furthermore, in the leave-one-out sensitivity analyses, excluding any individual study did not influence the overall findings (Supplementary Tables S1–S5).

### 3.3. Characterization of Methodological Approaches Across the Included Studies

#### 3.3.1. Summary LC *n*-3 PUFA Supplementation Protocols

Out of 43 studies, the majority examined supplementation with a combination of EPA + DHA, typically provided as triglycerides in a capsule-based LC *n*-3 PUFA supplement (Table 1). Only one study administered isolated EPA but did not specify if it was a methyl or ethyl ester [37], while another study investigated isolated DHA as a methyl ester [40]. Among the EPA + DHA studies, most utilized EPA dominant [28,29,31,35,38,41,46,47,48,49,53,54,55,58,59,60,61,62,63,64,65,66,67,70], four supplied DHA dominant [30,43,45,69], two provided equal ratios of EPA and DHA [51,52], and three did not specify the relative EPA and DHA composition [39,42,68] (Table 2).

The supplementation dosing strategies for combined doses of EPA + DHA ranged from 18.8 mg/day to 6400 mg/day [48,54]. The supplementation period ranged from 0 (same-day supplementation) to 70 days [48,57]. The majority of studies delivered LC *n*-3 PUFAs in capsule form, whereas three studies used beverages enriched with LC *n*-3 PUFA oil [51,52,69], and the method of administration was not specified in the remaining four studies [33,39,43,64]. The source of LC *n*-3 PUFAs was most commonly fish derived (27 studies), although some studies used alternative sources, including algae oil [45], krill oil [67], green-lipped mussel extract [44], and a combination of green-lipped mussel extract with krill oil [54]. Thirteen studies did not specify the source [30,31,35,37,38,40,42,57,62,63,64,68,69]. Nearly all studies reported the brand of fish oil used, and among those providing placebo details, oil-based formulations of vegetable oils, such as vegetable oil mixes or olive oil, were most frequently utilized for the placebo (Table 3).

Overall, 18 studies included a biological assessment of LC *n*-3 PUFA status [i.e., O3I or EPA/DHA levels] in response to supplementation. The most common approach was measuring the LC *n*-3 PUFA levels in whole blood from venous samples [28,31,50,51,52,61,66]; however, Ochi et al. (2017) [50] did not report the method of blood collection and reported only the results. Other approaches assessed the LC *n*-3 PUFA levels in plasma [32,41], serum [29,40,47,53,56], or neutrophil membranes [36]. Two studies measured the O3I (%EPA + %DHA in erythrocytes) [68,70], and one reported the O3I alongside the whole blood concentration of individual LC *n*-3 PUFAs [54]. To evaluate compliance, 27 studies used at least one compliance method: pill counting was the most common, while some relied solely on self-reporting (Table 4).

#### 3.3.2. Assessment of Participant Characteristics

Among the 43 included studies, most cohorts recruited male participants (*n* = 31), while fewer recruited both male and female participants (*n* = 7) or female participants only (*n* = 5) (Table 5). The groups’ mean participant ages ranged from 18.6 to 37.0 years, with most cohorts composed of healthy adults with varying training statuses (Tier 0 (58%), Tier 1 (28%), Tier 2 (9.3%), Tier 3 (2.3%), and Tier 4 (2.3%)) and body mass indexes (BMI—kg/m^2^) between 17.90 and 28.38 kg/m^2^. The sample sizes ranged from 8 to 64, with only 15 studies conducting a power calculation to determine the appropriate sample size required to detect significance for the primary outcome measure [27,31,38,39,44,45,47,51,52,58,60,65,66,67,68] (Table 5).

#### 3.3.3. Assessment of Exercise Models

Three studies were not specifically designed to induce muscle damage but were included in our analysis due to their relevant outcomes. These studies were Toft et al. (2000) [28], which measured pre- and post-marathon responses; Black et al. (2018) [51], who examined pre- and post-season responses in rugby players; and Buonocore et al. (2020) [55], who investigated pre- and post-exercise responses in both athlete and sedentary groups. The remaining 40 studies used EIMD protocols. The exercise models used to induce muscle damage varied dramatically across the studies (Table 1). Among the studies that induced muscle damage, resistance-based models were most commonly used, whereas fewer studies employed endurance or combined endurance–resistance protocols (Table 6). Within the resistance paradigms, the most frequently applied approaches were eccentric-only and combined eccentric–concentric, while a small number reported machine-free eccentric exercise or resistance training with unspecified details. In terms of targeted muscle groups, lower limb models were widely used, primarily targeting the hamstrings or quadriceps. Other studies concentrated on the upper body, specifically the elbow flexors. Additionally, one study examined both the upper and lower body [70], while two others did not specify the muscle group investigated [48,63] (Table 6).

### 3.4. Assessment of Muscle Damage Recovery Outcomes

#### 3.4.1. DOMS

DOMS was assessed in most of the included studies, with the majority using variations of a 100 mm Visual Analog Scale (VAS) at time points from immediately post-exercise to five days post-exercise (Table 7). However, five studies used different pain assessment scales: Tartibian et al. (2009) employed a 0–6 Talag scale [34]; Houghton and Onambele (2012) utilized the RPE Borg pain scale [37]; Black et al. (2018) used a 5-point Likert scale [51]; Asjodi et al. (2023) assessed DOMS with an unspecified pain scale [64]; and Makaje et al. (2024) applied a numeric pain rating scale [70]. Taken together, LC *n*-3 PUFA supplementation was frequently associated with significantly reduced DOMS severity, although several studies reported no effect, and one reported an increase [58] (Table 7).

#### 3.4.2. Muscle Damage Biomarkers

Muscle damage biomarkers, specifically CK, LDH, and/or Mb, were reported in most of the included studies, with collection times ranging from immediately post-exercise to 5 days later (Table 1). Notably, CK was the most frequently measured, either alone or with LDH and Mb, while only one study assessed Mb alone [49], and none measured LDH alone. LC *n*-3 PUFA supplementation was generally associated with significantly reduced levels of at least one biomarker, though several studies reported no effect, and one study observed an increase in Mb compared to placebo [49] (Table 7).

### 3.5. Assessment of Muscle Function

Muscle function was assessed in just over half of the studies included, with measurements taken at various points ranging from immediately post-exercise to five days post-exercise (Table 1). The most reported markers of muscle function, in order of frequency, were range of motion (ROM), maximal voluntary contraction (MVC), peak power, jump performance, peak torque, and lower body strength.

The ROM outcomes showed mixed evidence. Some studies reported improvements with LC *n*-3 PUFA supplementation [39,44,47,50,53,54,61], while others noted decreases [29,34,62,68] or no significant effects [30,40,42] (Table 8).

The MVC results were similarly inconsistent. Five studies identified significant improvements after LC *n*-3 PUFA supplementation [39,47,50,54], whereas most found no effect [41,44,52,56,59,60,61], and one reported a decrease compared with placebo [58] (Table 8).

The jump performance outcomes varied across studies. A few studies demonstrated improvements with LC *n*-3 PUFA supplementation [48,68], one reported a decrease [51], and others observed no significant effect [58,62,69] (Table 8).

Peak power was assessed less frequently: one study demonstrated improvements with LC *n*-3 PUFA supplementation [68], whereas others found no significant effect compared with placebo [54,59] (Table 8).

Only two studies assessing muscle function measured the peak torque following a damage protocol. LC *n*-3 PUFA supplementation improved the peak torque following muscle damage in one study [67], but had no effect in the other [66] (Table 8).

In summary, LC *n*-3 PUFA supplementation significantly enhanced at least one dimension of muscle function after muscle damage or intensive exercise in 15 studies, with no effect in 18 studies and a negative effect in 4.

### 3.6. Assessment of Inflammatory and Oxidative Stress Markers

#### 3.6.1. Inflammatory Markers

Inflammatory and oxidative stress blood markers were reported in 30 of the 43 studies (69.7%), with samples collected at various time points ranging from immediately post-exercise to 5 days later (Table 1). The most commonly reported inflammatory markers, in order of frequency, included interleukin-6 (IL-6), tumor necrosis factor-α (TNF-α), C-reactive protein (CRP), interleukin-1ra (IL-1ra), interleukin-1β (IL-1β), interleukin-8 (IL-8), interleukin-2 (IL-2), and interleukin-4 (IL-4). The most commonly assessed oxidative stress markers included malondialdehyde (MDA), superoxide dismutase (SOD), catalase (CAT), glutathione peroxidase (GPx), thiobarbituric acid reactive substances (TBARS), and total antioxidant capacity (T-AOC).

Among these markers, IL-6 was the most frequently assessed inflammatory blood marker. Notably, LC *n*-3 PUFA supplementation significantly reduced IL-6 levels in several studies compared with placebo [30,43,47,57,65]; however, one study reported a significant increase [37], and no significant effect was observed in the remaining studies (Table 9).

TNF-α was another frequently evaluated inflammatory blood marker; some studies found that LC *n*-3 PUFA supplementation significantly reduced TNF-α compared with placebo [31,36,44,54,55,63], whereas others found no significant changes (Table 9).

CRP was measured in about half of the studies assessing inflammatory blood markers; LC *n*-3 PUFA supplementation significantly reduced CRP levels following muscle damage in a few studies compared with placebo [30,31,38,42,65], whereas the remaining studies showed no significant changes (Table 9).

IL-1ra was assessed in a small number of studies evaluating inflammatory blood markers; LC *n*-3 PUFA supplementation reduced IL-1ra in one study compared with placebo [43], whereas no effect was observed in the remaining studies (Table 9).

IL-1β was rarely reported on among the included studies evaluating inflammatory blood markers; LC *n*-3 PUFA supplementation reduced IL-1β in one study compared with placebo [57], with no effect observed in the other studies [43,65] (Table 9).

IL-8 was evaluated in a limited number of studies on inflammatory blood markers; LC *n*-3 PUFA supplementation increased IL-8 in one study compared with placebo (33.3%) [43], whereas no effect was observed in the others [32,57] (Table 9).

Finally, IL-2 and IL-4 were each assessed in individual studies, both reporting no significant changes in these markers with LC *n*-3 PUFA supplementation compared to placebo [43,67] (Table 9).

#### 3.6.2. Oxidative Stress Markers

MDA was the most frequently reported oxidative stress marker among the studies assessing oxidative stress; LC *n*-3 PUFA supplementation significantly reduced MDA levels in several studies compared with placebo (50%) [38,55,67], whereas other studies reported no significant difference [29,31,33] (Table 10), highlighting the inconsistent results.

SOD was another commonly assessed oxidative stress marker among the included studies; LC *n*-3 PUFA supplementation increased SOD activity in three studies compared with placebo [33,49,67], whereas the remaining studies showed no effect [55] (Table 10).

Likewise, a few studies reported a significant increase in CAT levels with LC *n*-3 PUFA supplementation compared to placebo [33,55] (Table 10).

GPx was assessed in a few studies of oxidative stress markers. LC *n*-3 PUFA supplementation increased GPx activity in one study compared with placebo [55], while another found no effect [33] (Table 10).

Furthermore, one study on oxidative stress markers reported a significant increase in T-AOC levels with LC *n*-3 PUFA supplementation compared with placebo [67]. Similarly, another study found a significant increase in TBARS levels [41] (Table 10).

#### 3.6.3. Direct Measure of Swelling

Seven out of 30 studies (23%) assessed direct indicators of muscle inflammation using outcome measures such as upper arm circumference (UAC) [29,35,45,47,50,61], muscle thickness assessed by ultrasound [61], and muscle stiffness measured by elastography [45,53]. Six of these studies reported no significant change in UAC following supplementation, suggesting that LC *n*-3 PUFAs had little or no effect on muscle swelling in most cases. Similarly, Corder et al. (2016) [45] and Tsuchiya et al. (2021) [61] found no significant changes in muscle stiffness or thickness, supporting the finding of limited impact on direct inflammation markers. However, one study observed reductions in both UAC and muscle stiffness, indicating that, in this case, the anti-inflammatory effect of LC *n*-3 PUFA supplementation may have been sufficient to reduce swelling [43] (Table 11).

### 3.7. Meta-Analysis Results for DOMS, Muscle Strength, ROM, CK, and Swelling

#### 3.7.1. DOMS Meta-Analysis

**Figure 2:**
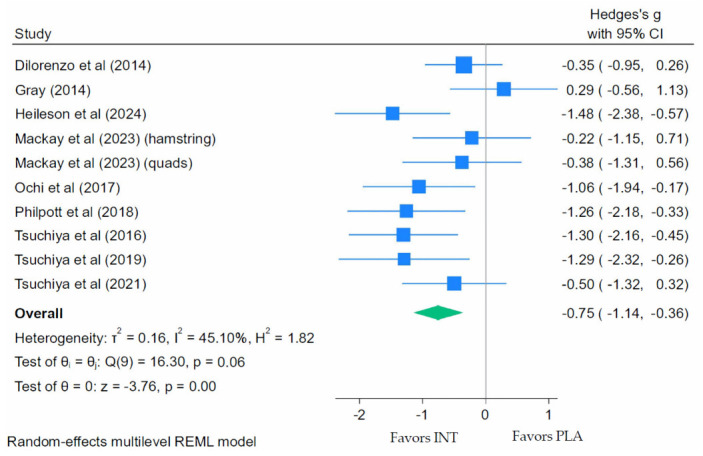
Forest plot of delayed onset muscle soreness (DOMS) at peak pain following eccentric exercise [40,41,47,50,52,53,61,66,68].

A meta-analysis of nine placebo-controlled eccentric exercise studies found that LC *n*-3 PUFA supplementation significantly reduced the VAS scores for DOMS, where the peak pain was recorded to occur between 24 and 72 h post-exercise compared with placebo (Figure 2). The pooled random-effects REML analysis showed a moderate-to-large effect (Hedges’ g = −0.75; 95% CI: −1.14 to −0.36; *p* < 0.001). Moderate heterogeneity was present (τ^2^ = 0.16; Q(9) = 16.30; *p* = 0.06; *I*^2^ = 45.10%; H^2^ = 1.82).

#### 3.7.2. CK Meta-Analysis

**Figure 3:**
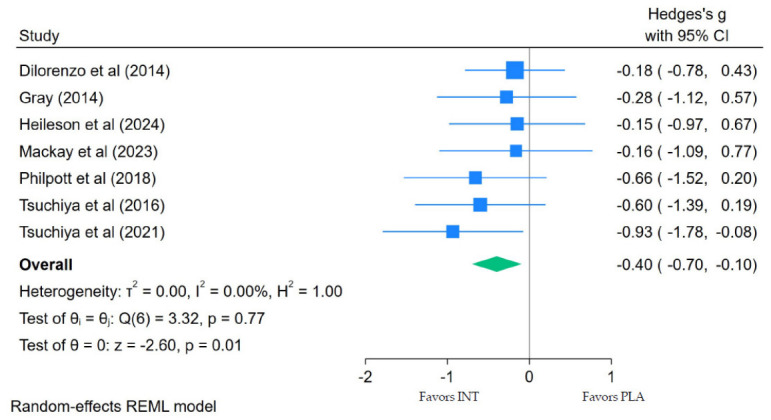
Forest plot of creatine kinase (CK) at peak concentration following eccentric exercise [40,41,47,50,52,53,61,66,68].

CK was measured in seven of the nine studies we identified. The meta-analyses showed that LC *n*-3 PUFA supplementation significantly reduced CK levels following EIMD when compared with placebo. Specifically, a pooled analysis for CK (Figure 3) found a small effect (Hedges’ g = −0.40; 95% CI: −0.70 to −0.10; *p* = 0.01), with no heterogeneity (τ^2^ = 0.00; *I*^2^ = 0.00%; Q(6) = 3.32; *p* = 0.77).

#### 3.7.3. Muscle Strength Meta-Analysis

**Figure 4:**
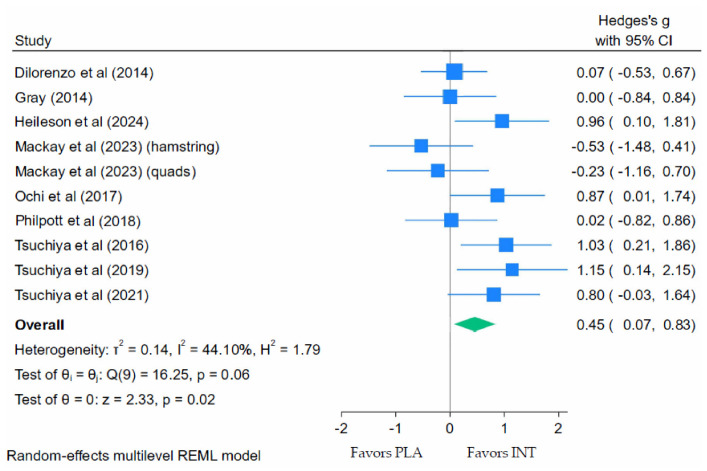
Forest plot of muscle strength at peak strength loss following eccentric exercise [40,41,47,52,61,66,68].

Meta-analyses of strength data extracted from the nine studies revealed that LC *n*-3 PUFAs significantly improved muscle strength during recovery from EIMD compared with placebo (Figure 4). The pooled results indicated a small-to-moderate effect (Hedges’ g = 0.45; 95% CI: 0.07 to 0.83; *p* = 0.02), with moderate heterogeneity (τ^2^ = 0.14; *I*^2^ = 44.10%; Q(9) = 16.25; *p* = 0.06).

#### 3.7.4. ROM Meta-Analysis

**Figure 5:**
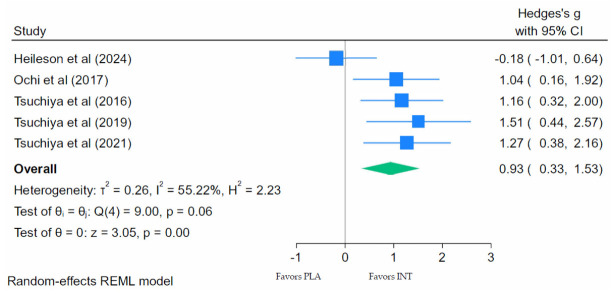
Forest plot of range of motion (ROM) at peak reduction following eccentric exercise [47,50,53,61,68].

ROM was assessed in five of the nine studies we identified. The meta-analysis of the extracted data demonstrated significantly improved ROM following EIMD when compared with placebo (Figure 5; Hedges’ g = 0.93; 95% CI: 0.33 to 1.53; *p* < 0.001), indicating a large effect. Moderate heterogeneity was observed for the ROM outcomes, indicating variability among the study results (τ^2^ = 0.26; *I*^2^ = 55.22%; Q(4) = 9.00; *p* = 0.06).

#### 3.7.5. Muscle Swelling Meta-Analysis

**Figure 6:**
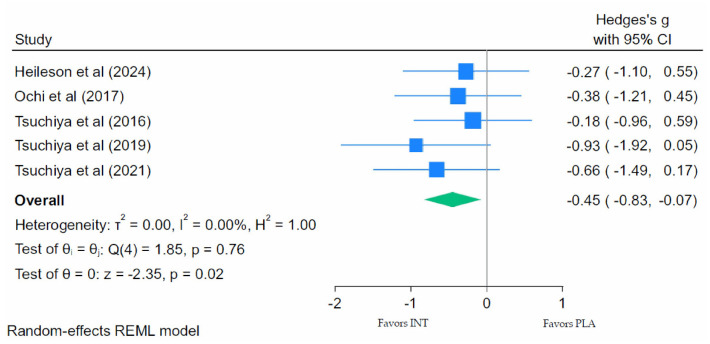
Forest plot of muscle swelling at peak swelling following eccentric exercise [47,50,53,61,68].

Muscle swelling was assessed via limb circumference or ultrasound thickness in five of the nine studies identified for meta-analysis. Muscle swelling following EIMD was significantly reduced (Figure 6; Hedges’ g = −0.45; 95% CI: −0.83 to −0.07; *p* = 0.02), indicating a small-to-moderate effect of LC *n*-3 PUFA supplementation compared with placebo supplementation. There was no heterogeneity for the muscle swelling outcomes (τ^2^ = 0.00; *I*^2^ = 0.00%; Q(4) = 1.85; *p* = 0.76), indicating consistency in the effects across studies.

## 4. Discussion

This systematic review and meta-analysis provides a comprehensive evaluation of the effects of LC *n*-3 PUFA supplementation on post-exercise recovery from eccentric/ muscle-damaging exercise in healthy adults, encompassing a broad range of outcomes, including muscle soreness, muscle damage biomarkers, muscle function, inflammation, and oxidative stress, across diverse exercise models and participant populations. The Cochrane risk of bias assessment indicates that many studies exhibited a moderate-to-high risk of bias, primarily due to issues of study design, conduct, analysis, and reporting. These methodological concerns mirror those highlighted by Anthony et al. [75,76], and should be considered when interpreting our findings. Due to the high levels of heterogeneity across study designs and inconsistency in study quality, the systematic review portion of this analysis suggests that supplementation with LC *n*-3 PUFAs produces equivocal effects on recovery outcomes for eccentric/muscle-damaging exercise models. Furthermore, the lack of consistency in dosing strategies prevents assessments of dose/duration thresholds necessary to achieve a beneficial effect. In the meta-analysis portion of this review, nine studies with similar designs (all eccentric-induced muscle damage) and confirmed improvements in LC *n*-3 PUFA status following supplementation were included. Our selection criteria were set to find studies that we felt would be sufficiently similar in design with adequate quality controls to produce what we hope is a reliable meta-analysis. However, we must preface the results with the following considerations: four of the nine studies [47,50,53,61] are from the same lab, a further two studies are from the same lab [52,66], and the studies include a combination of lower limb and upper limb muscle groups.

This analysis demonstrates that LC *n*-3 PUFA supplementation significantly improved muscle damage recovery outcomes, such as DOMS, maximum force, ROM, and CK, as well as direct measures of muscle swelling, including limb circumference/muscle thickness. Previous systematic reviews and meta-analyses [20,22,23,77] have been focused and relatively narrow. In contrast, the present review uses a broader, more integrated approach, incorporating evidence from across multiple physiological domains, exercise interventions, and supplementation strategies.

### 4.1. LC n-3 PUFA Supplementation Design Issues

Compliance assessment remains a critical methodological limitation across the studies in this review. Although many trials described the intended supplementation protocols, only 18 studies objectively assessed adherence using biological markers. Of the studies that assessed compliance using biological markers, seven relied on plasma or serum fatty acid concentrations, which are sensitive to short-term dietary intake and may not accurately reflect longer-term incorporation of LC *n*-3 PUFAs into tissues [29,32,40,41,47,53,56]. In contrast, the O3I, which measures EPA and DHA levels in red blood cell membranes or estimates from dried blood spots [78], provides a stable and validated marker of long-term LC *n*-3 PUFA status and offers a more robust approach to long-term compliance assessment. Only three studies assessed the O3I [54,68,70]. Both plasma or serum fatty acid levels and the O3I represent objective methods of compliance assessment, but their inconsistent and limited use across trials restricts the ability to verify whether the supplementation protocols achieved meaningful biological incorporation of LC *n*-3 PUFAs into the target tissues.

The proposed mechanism of LC *n*-3 PUFA supplementation is through the incorporation of EPA and DHA into cell membranes, reducing the proportion of omega-6 fatty acids, including arachidonic acid (AA; 20:4*n*-6). Theoretically, this would mean that less AA is available for the production of pro-inflammatory molecules [79,80], such as prostaglandins and leukotrienes [81]. This change in muscle membrane fatty acid composition may reduce the production of pro-inflammatory mediators and potentially attenuate DOMS, inflammation, and muscle damage after eccentric exercise [82]. This highlights the need for future studies to incorporate systemic or tissue-based biomarker compliance assessments, ideally including the O3I at baseline and post-intervention, to strengthen the reliability of findings.

Another important methodological limitation of the study designs is the lack of distinction between the specific types of LC *n*-3 PUFAs administered, as most studies provided supplements containing a mixture of EPA and DHA. Only three studies supplemented with either EPA or DHA in isolation [30,37,40]. DHA is the primary LC *n*-3 PUFA in the membranes of cardiac [83] and skeletal muscle cells [82,84], and it is preferentially incorporated into these membranes, whether a supplement is rich in DHA or EPA [83,84]. Therefore, it may be beneficial to assess direct tissue incorporation via muscle biopsies or, at a minimum, use indirect blood-based markers to evaluate EPA and DHA levels. This would enable specific conclusions to be drawn about the effectiveness of EPA relative to DHA at not just shifting the fatty acid profile of tissues, but also to determine if there are differential effects of EPA vs DHA on recovery outcomes. That said, one study [48] found a positive effect of acute (same day of damage protocol) supplementation on EIMD recovery outcomes. These data suggest that tissue incorporation of LC *n*-3 PUFAs may not be required for recovery outcomes, and so additional, acute (same day) protocols are also needed to confirm this.

### 4.2. Effects of LC n-3 PUFAs on DOMS

DOMS can be assessed by a range of different methods, usually subjective questionnaire-based protocols performed during rest, exercise or in various body positions with or without palpation. Most studies in this review used a 100 mm VAS to assess pain intensity, where mild pain was defined as 5–44 mm, moderate pain as 45–74 mm, and severe pain as 75–100 mm [85]. However, few studies specified their pain assessment standardization procedures. This notwithstanding, and taking the DOMS data at face value, the majority of studies reported that LC *n*-3 PUFA supplementation reduced DOMS severity compared to placebo. For instance, 19 out of 32 studies (59%) supported this effect [34,35,39,42,44,45,47,50,51,52,53,54,57,58,59,63,64,68,70], and one study (3%) reported negative effects of LC *n*-3 PUFA supplementation [49]. It is noteworthy that the limited number of studies assessing the O3I (control group mean O3I = 5.40% and intervention group mean O3I = 7.67%) also demonstrated reductions in DOMS severity following LC *n*-3 PUFA supplementation [54,68,70]. These three studies were rated as having a low risk of bias and had intervention durations of approximately 30 days or longer, which would provide sufficient time for the LC *n*-3 PUFA profile of the muscle to change [11]. However, the limited number of such studies highlights the need for further high-quality research to confirm these observations.

A key goal of this systematic literature review was to assess if we could define threshold doses/durations to achieve a positive outcome of supplementation. However, determining the effective dose and supplementation duration for reducing DOMS remains challenging, as both short-term (7–21 days) and long-term (≥30 days) protocols, as well as low (18.8–3000 mg/day) and high (4000–6000 mg/day) doses, have demonstrated beneficial effects on recovery outcomes [20,22,77]. We hypothesize that similar outcomes with different dosing strategies may have resulted from a similar O3I status, and it may also be possible that LC *n*-3 PUFA supplementation may have an acute effect without the requirement for long-term supplementation to change a tissue’s fatty acid composition. However, differentiating these effects is currently not feasible because so few studies have measured LC *n*-3 PUFA status and there are not sufficient studies using similar dose/duration strategies to determine these thresholds.

Whilst the minimally effective dose/duration variables for LC *n*-3 PUFA dosing cannot be defined, one factor was identified that may cluster with positive outcomes. This is the degree of pain achieved by the EIMD protocol. Among the 19 studies reporting positive effects of LC *n*-3 PUFA supplementation, 12 involved participants who experienced moderate-to-severe pain (45–74 mm and 75–100 mm) following the muscle-damaging exercise stimulus. In contrast, among the 12 studies reporting null effects, 11 described pain levels ranging from mild to moderate. These data potentially suggest that detecting an effect of LC *n*-3 PUFA supplementation requires at least moderate (45–74 mm) levels of pain to be achieved by the damage protocol. Our meta-analysis, which includes studies with moderate–severe levels of pain, demonstrates an overall effect favoring LC *n*-3 PUFA supplementation for reducing perceived DOMS following EIMD. While the effect sizes varied, the pooled results indicate that LC *n*-3 PUFA supplementation significantly reduced DOMS during post-exercise recovery from EIMD.

Taken together, the pooled and narrative evidence supports the effectiveness of LC *n*-3 PUFA supplementation for reducing perceived muscle soreness following EIMD, with benefits observed across a wide range of doses (18.8–6000 mg/day). Furthermore, the evidence suggests that the probability of finding a positive effect of LC *n*-3 PUFA supplementation increases when the muscle damage or exercise model induces moderate-to-severe pain (45 mm+ on a 100 mm VAS).

### 4.3. Effects of LC n-3 PUFAs on Muscle Damage Biomarkers

Muscle damage biomarkers provide some insight into the extent of muscle fiber disruption and thus into the potential protective effects of LC *n*-3 PUFA supplementation during recovery. Among the circulating markers assessed, CK was measured most frequently, with some studies also measuring LDH and Mb to evaluate muscle damage. Of the 31 studies measuring CK, 13 (42%) reported significant reductions in post-exercise CK concentrations following LC *n*-3 PUFA supplementation. Among these 13, five studies (38.4%) included participants with moderate-to-severe pain. Notably, 12 (66.6%) of the 18 studies that reported no significant changes in muscle damage biomarkers involved participants who experienced only mild-to-moderate muscle soreness. Similar to the DOMS outcomes, it may be that detecting an effect of LC *n*-3 PUFA supplementation may be more likely for CK if moderate–severe soreness is achieved by the protocol.

Of the nine studies that we selected for the meta-analysis on DOMS, seven of those measured CK. A quantitative synthesis of these studies, including four with participants experiencing moderate-to-severe pain, showed an overall effect in favor of LC *n*-3 PUFA supplementation attenuating post-exercise CK responses. These findings provide preliminary support for the thesis that LC *n*-3 PUFAs may reduce muscle damage and limit the leakage of intracellular enzymes such as CK [20].

However, the above conclusion needs to be tempered by the following considerations: (1) CK responses vary significantly among individuals and are influenced by other factors, such as muscle mass, training history, and genetic predisposition, resulting in high inter-individual variability and sensitivity to prior training [79,86]. Therefore, the reliability of CK as a biomarker of muscle damage remains debated [79,86]. (2) Positive outcomes for other recovery markers do not always co-occur with positive outcomes for CK. For instance, Lembke et al. (2014) found no significant changes in CK levels after LC *n*-3 PUFA supplementation, even when other recovery outcomes significantly improved [42]. So, while the pooled evidence indicates LC *n*-3 PUFA supplementation can reduce damage-induced increases in CK, an assessment of CK alone is likely insufficient to confirm protection against muscle damage. Therefore, blood CK assessments should be complemented by functional and perceptual evaluations to comprehensively assess the impact of LC *n*-3 PUFAs on EIMD.

### 4.4. Effects of LC n-3 PUFAs on Muscle Function

The effect of LC *n*-3 PUFA supplementation on muscle function following EIMD has been investigated using various performance-related outcomes. Of the 25 studies assessing functional recovery, 15 reported beneficial effects of LC *n*-3 PUFA supplementation, with improvements most commonly observed in strength, power, and overall functional performance within 48–96 h post-exercise. For instance, Tsuchiya et al. (2016) found that eight weeks of LC *n*-3 PUFA supplementation (600 mg/day EPA + 260 mg/day DHA) significantly accelerated the recovery of MVC and elbow range of motion following eccentric bicep exercises [47]. Similarly, Black et al. (2018) reported that five weeks of supplementation (approximately 1100 mg/day of EPA + DHA) during the rugby season enhanced the post-season vertical jump performance of rugby athletes compared to those who received a placebo [51]. However, not all studies have demonstrated positive effects, and these discrepancies cannot be explained by differences in dose/duration protocols, but may be due to differences in the exercise models and methods of functional assessment. The quantitative synthesis we completed during the meta-analysis suggests that LC *n*-3 PUFA supplementation can significantly improve muscle functional recovery following EIMD. The pooled estimates show a small-to-moderate improvement in muscle strength with LC *n*-3 PUFA supplementation at peak dysfunction.

Another assessment of muscle function employed across five of the nine studies in our meta-analysis was a ROM assessment. ROM is often limited following a muscle damage stimulus and provides an alternative dimension of functional recovery distinct from force production. The pooled analysis of ROM outcomes across these studies reveals a large, significant improvement following LC *n*-3 PUFA supplementation, indicating accelerated recovery of joint mobility after muscle-damaging exercise. Although the individual study effects varied, the overall pooled evidence suggests that LC *n*-3 PUFA supplementation facilitates functional recovery by supporting both strength restoration and the recovery of movement capacity during the post-exercise period. However, these conclusions must again be tempered by the fact that two labs contributed six of the nine studies on strength analysis and one lab contributed four of the five studies on ROM analysis.

### 4.5. Effects of LC n-3 PUFAs on Inflammation and Oxidative Stress

LC *n*-3 PUFAs have the ability to be incorporated into cell membranes, including skeletal muscle [11] and mitochondrial membranes [80], and regulate the synthesis of lipid mediators that modulate inflammatory processes [20,81]. As a result, LC *n*-3 PUFAs have the capacity to modulate oxidative stress through regulating mitochondrial function, membrane integrity and inflammatory pathways. Therefore, it is reasonable to assume that the effects of EIMD, which generates a robust oxidative stress [87] and inflammatory response [5], may be moderated by LC *n*-3 PUFA incorporation into muscle. If this mechanism is at play, then it would be reasonable to hypothesize that muscle swelling indicators may be reduced by LC *n*-3 PUFA supplementation following EIMD.

Of the nine studies that we selected for the meta-analysis, five made a pre–post measure of muscle swelling (limb circumference or thickness via ultrasound). Consistent with the proposed mechanistic rationale, we found a small-to-moderate but significant reduction in EIMD-induced markers of swelling following LC *n*-3 PUFA supplementation. However, it is important to note that the pooled studies came from only two labs; the absence of heterogeneity may be attributable to the limited number of labs testing this outcome. Being mindful of the limitations regarding muscle swelling assessment, these results provide some support for the proposed anti-inflammatory properties of LC *n*-3 PUFAs in the context of EIMD and suggest that supplementation may help reduce muscle swelling and subsequent tissue damage following EIMD. However, to our knowledge, no studies have assessed this effect directly in human muscle with biopsy approaches.

If the proposed model of LC *n*-3 PUFA action is correct, then circulating markers of inflammation would also be suppressed by LC *n*-3 PUFA supplementation following EIMD. However, the evidence concerning circulating inflammatory and oxidative stress markers is highly variable. Although inflammation and oxidative stress are secondary outcomes of this review, they are central to the proposed mechanism of action of LC *n*-3 PUFA supplementation. Overall, the evidence indicates a weak link between LC *n*-3 PUFA supplementation and changes in inflammation or oxidative stress. While the pooled analyses suggest significant effects of LC *n*-3 PUFA supplementation on the EIMD response (DOMS, function, CK, and swelling), individual studies often show discordant responses—reductions in DOMS do not consistently coincide with reductions in other markers, and vice versa. Moreover, in our assessment of the literature to this point, the available data is insufficient to conduct correlational analyses across outcomes to formally assess mechanistic coupling.

## 5. Limitations

Several methodological limitations must be considered when interpreting this body of literature and our meta-analysis. Many of the studies included did not report how the sample sizes were determined, and power calculations justifying the sample sizes were rarely included. Heterogeneity of participant characteristics, such as training status, and the absence of female participants were also evident. Of most concern was the variability in the supplementation protocols, including differences in dosing strategies and intervention durations. Differences in the outcome measures (e.g., different pain assessment tools or tools that were not described), combined with a lack of compliance testing and objective markers of supplementation success (e.g., O3I) and variation in the exercise protocols implemented, further limited our ability to determine effective dosing strategies. Furthermore, whilst our meta-analysis suggests significant improvements in outcomes such as DOMS, CK, function, and swelling, we must temper the results with the following considerations: (1) four of the nine studies [47,50,53,61] are from the same lab, a further two studies are from the same lab [52,66], and (2) the studies include a combination of lower limb and upper limb muscle groups. Finally, there is a lack of “minimal clinically important difference” thresholds for many of these outcomes in the context of EIMD. So, whilst there are significant effects shown from the meta-analysis, it is unclear if the magnitude of these effects would translate into clinical, physiological or competitively meaningful outcomes.

In this context our findings reflect those of Anthony et al. (2024) [76], who carried out a related review focused on physically trained participants, and Anthony et al. (2021) [75], who reviewed the effects of LC *n*-3 PUFAs on eccentric exercise protocols with DOMS and inflammation as outcomes. Both systematic reviews highlighted consistent design flaws across the literature base. Interestingly, Anthony et al. (2024) employed a custom 5-point quality assessment scale specifically for this area of research, and none of the studies they analyzed satisfied all five quality recommendations [76].

The other limiting factor in this field is the diversity of commercially available LC *n*-3 PUFA products, in addition to the availability of LC *n*-3 PUFAs in food sources. With practitioners or individuals in mind, the state of the current literature base makes it impossible to suggest a specific dose/duration/source for positive outcomes. The primary thesis for the biological action of LC *n*-3 PUFAs is via incorporation into tissue membranes where they can influence membrane fluidity, oxidative stress and inflammatory signaling. Thus, as is part of the custom 5-point assessment tool employed by Anthony et al. (2024), all future research in this space should measure LC *n*-3 PUFA incorporation into tissues with blood markers, such as the O3I as the minimum standard [76]. With sufficient O3I (or similar) data, it may then be possible to bypass dosing/sourcing questions and make recommendations based on a set O3I or another marker.

Whilst it might benefit individual companies to develop a product claim for their specific products with dose and duration protocols from placebo-controlled RCTs, this does not benefit a practitioner or individual user who may not be able to access (due to cost/location, etc.) or use (due to allergies or aversions to fish) a specific product. However, knowing the degree to which LC *n*-3 PUFAs are incorporated into tissues may help with this decision making, providing a validated test is accessible.

## 6. Future Perspectives

In future research, several priorities should be addressed to strengthen the evidence base. First, studies should be powered to detect the level of changes at which reductions in muscle soreness or related recovery outcomes become practically meaningful (minimal clinically important difference, smallest worthwhile change, etc.), rather than focusing only on statistical significance. Second, more research is needed on under-represented populations, particularly trained participants and female athletes, to improve the generalizability of findings. Third, mechanistic studies are required to clarify how LC *n*-3 PUFAs may influence recovery, including whether their effects are mediated through acute responses to circulating LC *n*-3 PUFAs, or if altered membrane composition from long-term supplementation is required to alter inflammation, neuromuscular function, or other pathways. In this context, studies should also seek to isolate the effects of EPA and DHA and, where relevant, examine whether the relative ratio of these fatty acids influences outcomes. Finally, future trials should incorporate objective measures of LC *n*-3 PUFA status, with the O3I representing a particularly useful tool because it is accessible, relatively inexpensive, standardized, validated against red blood cell fatty acids, and widely used. Applying the O3I would allow studies to confirm biological exposure, improve comparability across protocols, and help determine whether a threshold of tissue incorporation is required for recovery benefits. Over time, this may enable more precise and transferable recommendations based on achieved omega-3 status, rather than being limited to a specific supplement brand, source, formulation, dose, or duration.

## 7. Conclusions

While methodological limitations, including heterogeneity of study designs, small sample sizes, and inconsistent compliance reporting, limit the certainty of our conclusions, the available evidence tentatively supports LC *n*-3 PUFA supplementation as a non-pharmacological strategy to assist recovery from EIMD. The most consistent signal was observed for reduced muscle soreness, with more limited evidence for benefits to strength and functional recovery outcomes. However, the current evidence base is not yet sufficient to support definitive recommendations regarding optimal dose, duration, or target omega-3 status.

## Figures and Tables

**Table 1:** Summary of included studies and extracted data. Tier 0 = sedentary; Tier 1 = recreationally active; Tier 2 = trained/developmental; Tier 3 = highly trained/national level; Tier 4 = elite/international level; Tier 5 = world class. Abbreviations: INT = intervention group (received LC *n*-3 PUFA supplement); PLA = placebo group (received a placebo treatment); CON = control group (no active or placebo treatment); EPA = eicosapentaenoic acid; DHA = docosahexaenoic acid; DPA = docosapentaenoic acid; DOMS = delayed onset muscle soreness; CK = creatine kinase; LDH = lactate dehydrogenase; Mb = myoglobin; PUFA = polyunsaturated fatty acid; ROM = range of motion; MVC = maximal voluntary contraction; IL-6 = interleukin-6; TNF-α = tumor necrosis factor-α; CRP = C-reactive protein; IL-1ra = interleukin-1ra; IL-1β = interleukin-1β; IL-8 = interleukin-8; IL-2 = interleukin-2; IL-4 = interleukin-4; MDA = malondialdehyde; SOD = superoxide dismutase; CAT = catalase; GPx = glutathione peroxidase; TBARS = thiobarbituric acid reactive substances; T-AOC = total antioxidant capacity; UAC = upper arm circumference; +ve = positive; −ve = negative; ECC = eccentric contraction.

Authors (Year)	Participant Details	Supplement Details	Compliance Method	Muscle Damage Model/Mode of Exercise	Outcome Measures	Pain Level	LC *n*-3 PUFA Change	Primary Findings	Effect
Toft et al. (2000) [28]	Trained runners Tier 2 Males (*n* = 20) **Age (Mean ± SD)**: INT: 29 ± 6.25; CON: 28 ± 4.75 years **INT**: *n* = 10 **CON:** *n* = 10	**Mass of oil (mg)**: 6000 **EPA + DHA + DPA (mg)**: 3600 **Brand**: Pikasol, Lube **Placebo**: No data **Supplement period (days):** 42	No data	The Copenhagen Marathon 1998	CK IL-6 IL-1ra TNF-α	No data	Blood EPA and DHA significantly increased in INT group; no changes in control group	**All outcomes measures:** No significant change	No effect
Lenn et al. (2002) [29]	Untrained Tier 0 Males/Females (*n* = 16) **Age (Mean ± SD)**: M: 22.7 ± 3.92); F: 24.5 ± 5.47 years **INT**: *n* = 5 **PLA**: *n* = 5	**Mass of oil (mg)**: 1800 **EPA + DHA + DPA (mg)**: 714.6 **Brand**: No data **Placebo**: Western fat blend and/or wheat flour **Supplement period (days)**: 37	No data	50 maximal effort eccentric contractions of the non-dominant arm at 90 °/s	DOMS ROM CK MDA IL-6 TNF-α UAC	Mild pain	Serum content of EPA and DHA increased by approximately 4-fold in INT group; no changes in control groups	**ROM:** Significant reduction (INT at 48 h & 96 h post damage) **All other outcomes measures:** No significant change	−ve (ROM)
Phillips et al. (2003) [30]	Untrained Tier 0 Males (*n* = 35) **Age (Mean ± SD)**: 22.1 ± 3.9 years **INT**: *n* = 16 **PLA**: *n* = 19	**Mass of oil (mg)**:1400 **EPA + DHA + DPA (mg)**: 800 **Brand**: Martek Biosciences, USA **Placebo**: Sunflower oil **Supplement period (days)**: 14	Pill counting	3 sets of 10 at 80% of eccentric 1RM on non-dominant arm; each repetition lasted 6 s; 2 min rest between sets	DOMS ROM LDH CK IL-6 CRP	Mild pain	No data	**IL-6 and CRP:** Significant reduction (INT at 72 h post damage) **All other outcomes measures:** No significant change	+ve (IL-6, CRP)
Bloomer et al. (2009) [31]	Recreationally active Tier 1 Males (*n* = 14) **Age (Mean ± SD)**: 25.5 ± 4.8 years **INT**: *n* = 14 **PLA**: *n* = 14	**Mass of oil (mg)**: No data **EPA + DHA + DPA (mg)**: 4432 **Brand**: Minami Nutrition, Belgium **Placebo**: Soybean **Supplement period (days)**: 42	Pill counting	Walking on a treadmill while carrying a weighted backpack (weight equal to 25% of body mass) for 60 min; the treadmill speed and grade were altered every five mins	DOMS CK CRP TNF-α MDA	Mild pain	Blood EPA and DHA significantly increased in INT group	**CRP and TNF-α:** Significant reduction (INT at rest post damage) **All other outcomes measures:** No significant change	+ve (CRP, TNF-α)
Nieman et al. (2009) [32]	Trained cyclists Tier 2 Males/Females (*n* = 23) **Age (Mean ± SD)**: INT: 24.1 ± 2.4; PLA: 26.9 ± 2.8 years **INT**: *n* = 11 **PLA**: *n* = 12	**Mass of oil (mg)**: No data **EPA + DHA + DPA (mg)**: 2400 **Brand**: The Cooper Aerobics Center, USA **Placebo**: Same as supplement without fish oil **Supplement period (days)**: 45	No data	Cycling for 3 h/d for 3 days at -57% m Wmax, with 10 km time trials inserted during the final 15 min of each 3 h bout	CK CRP IL-1ra IL-6 IL-8	No data	Plasma EPA (311%) and DHA (40%) significantly increased in INT group; no changes in control group	**All outcomes measures:** No significant change	No effect
Poprzecki et al. (2009) [33]	Recreationally active Tier 1 Males (*n* = 24) **Age (Mean ± SD)**: INT: 21.0 ± 90.9; PLA: 20.7 ± 91.1 years **INT**: *n* = 12 **PLA**: *n* = 12	**Mass of oil (mg)**: 1300 **EPA + DHA + DPA (mg)**: 650 **Brand**: Rybasol Pronova Biocare A/S, Norway **Placebo**: Gelatin **Supplement period (days)**: 42	No data	1 h cycloergometer test with a constant workload corresponding to 60% of VO2max and various pedaling rates: steady over the first 45 min (60 rev/min) and maximum over the last 15 min	CK MDA SOD CAT GPx	No data	No data	**CAT:** Significant increase (INT at 1 h post damage) **SOD:** Significant increase (INT immediately post damage) **All other outcomes measures:** No significant change	+ve (CAT, SOD)
Tartibian et al. (2009) [34]	Untrained Tier 0 Males (*n* = 27) **Age (Mean ± SD)**: 33.4 ± 4.2 years **INT**: *n* = 9 **PLA**: *n* = 9 **CON**: *n* = 9	**Mass of oil (mg)**: No data **EPA + DHA + DPA (mg)**: 540 **Brand**: Viva Pharmaceutical, Inc., Canada **Placebo**: No data **Supplement period (days)**: 32	No data	40 min of bench stepping, with 5 min stepping and 1 min rest between stepping periods	ROM	No data	No data	**ROM:** Significant reduction (INT at 48 h post damage)	−ve (ROM only)
Jouris et al. (2011) [35]	Untrained Tier 0 Males/Females (*n* = 11) **Age (Mean ± SD)**: 35 ± 10 years **Design: Within-subject crossover** **CON (14 days, low n-3 diet):** EIMD protocol **INT (7 days, n-3 supplement):** EIMD protocol	**Mass of oil (mg)**: No data **EPA + DHA + DPA (mg)**: 3000 **Brand**: Natural Factors, USA **Placebo**: Not Applicable **Supplement period (days)**: 7	Pill counting	Using 120% of the subject’s 1RM, 2 sets of eccentric biceps curls with 60 s of rest between sets	DOMS UAC	Moderate-to- severe pain	No data	**DOMS:** Significant reduction (INT at 48 h post damage) **All other outcomes measures:** No significant change	+ve (DOMS only)
Tartibian et al. (2011) [36]	Untrained Tier 0 Males (*n* = 45) **Age (Mean ± SD)**: 29.7 ± 6.6 years **INT**: *n* = 15 **PLA**: *n* = 15 **CON**: *n* = 15	**Mass of oil (mg)**: 1800 **EPA + DHA + DPA (mg)**: 540 **Brand**: Viva Pharmaceutical, Inc., Canada **Placebo**: Soybean/corn oil mix **Supplement period (days)**: 32	No data	50 cm stepping for 5 min with 1 min rest for 40 min, alternating leg every 10 min	LDH CK TNF-α Mb	No data	Neutrophil EPH and DHA content increased significantly in INT group; percentage not reported	**TNF-α and LDH:** Significant reduction (INT immediately and at 24 h & 48 h post damage) **IL-6, CK, and Mb:** Significant reduction (INT at 24 h & 48 h post damage)	+ve (TNF-α, CK, LDH, IL-6, Mb)
Houghton & Onambele (2012) [37]	Untrained Tier 0 Females (*n* = 17) **Age (Mean ± SD)**: 20.4 ± 2.1 years **INT**: *n* = 7 **PLA**: *n* = 10	**Mass of oil (mg)**: 2000 **EPA + DHA + DPA (mg)**: 360 **Brand**: MyProtein, UK **Placebo**: Lecithin **Supplement period (days)**: 21	No data	3 sets of 10 reps at 70% of 1RM of 4 exercises (leg extension, flexion, straight leg dead lifts, and walking lunges) over 45 min	IL-6 DOMS Muscle strength	No data	No data	**IL-6:** Significant increase (INT by the third set of eccentric workouts) **All other outcomes measures:** No significant change	−ve (IL-6 only)
Atashak et al. (2013) [38]	Handball players Tier 2 Males (*n* = 20) **Age (Mean ± SD)**: INT: 20.24 ± 1.87; PLA: 21.55 ± 2.34 years **INT**: *n* = 10 **PLA**: *n* = 10	**Mass of oil (mg)**: 3000 **EPA + DHA + DPA (mg)**: 900 **Brand**: Pty Ltd., Brookvale, Australia **Placebo**: Matched placebo capsule **Supplement period (days)**: 7	Self-reported	Resistance exercises: 3 leg exercises, including leg press, leg extension, and leg curls at 120% of the participants’ predicted 1RM for each exercise; The participants completed 40 reps (4 sets × 10, with 3 min rest between sets) of each exercise	CK LDH CRP MDA	No data	No data	**CRP, CK, and MDA:** Significant reduction (INT at 24 h post damage) **All other outcomes measures:** No significant change	+ve (CRP, CK, MDA)
Rajabi et al. (2013) [39]	Untrained Tier 0 Males (*n* = 20) **Age (Mean ± SD)**: 20.5 ± 1.8 years **INT**: *n* = 10 **PLA**: *n* = 10	**Mass of oil (mg)**: 2000 **EPA + DHA + DPA (mg)**: No data **Brand**: Viva *n*-3 Fish Oil, Canada **Placebo**: No data **Supplement period (days)**: 32	No data	4 sets of 20 reps of eccentric quadricep contractions at 75% 1RM using leg press machine	DOMS ROM MVC LDH CK	Moderate pain	No data	**DOMS:** Significant reduction (INT at 24 h, 48 h, & 72 h post damage) **MVC:** Significant increase (INT immediately and at 48 h & 72 h post damage) **ROM:** Significant increase (INT at 48 h & 72 h post damage) **CK and LDH:** Significant reduction (INT at 48 h & 72 h post damage)	+ve (DOMS, MVC, ROM, CK, LDH)
DiLorenzo et al. (2014) [40]	Untrained Tier 0 Males (*n* = 41) **Age (Mean ± SD)**: 21.8 ± 2.7 years **INT**: *n* = 21 **PLA**: *n* = 20	**Mass of oil (mg)**: No data **EPA + DHA + DPA (mg)**: 2000 **Brand**: Martek Biosciences Corporation, USA **Placebo**: Corn oil **Supplement period (days)**: 28	Pill counting	6 sets of 10 ECC bicep curls to failure at 140% of 1RM with 2 min rest between sets, followed by 5 gym sessions including 3–4 sets of 8 repetitions of each exercise	DOMS ROM IL-6 IL-1ra CRP CK	Mild pain	Serum levels of DHA increased 380% in INT group; no changes in control group	**CK:** Significant reduction (INT at 96 h post damage) **All other outcomes measures:** No significant change	+ve (CK only)
Gray et al. (2014) [41]	Untrained Tier 0 Males (*n* = 20) **Age (Mean ± SD)**: 23 ± 2.3 years **INT**: *n* = 10 **PLA**: *n* = 10	**Mass of oil (mg)**: 3000 **EPA + DHA + DPA (mg)**: 1600 **Brand**: Nordic Naturals **Placebo**: Olive oil **Supplement period (days)**: 42	No data	20 sets of 10 ECCs (knee flexion/extension) with 2 min rest between sets	DOMS MVC CK TBARS	Mild pain	Plasma concentration of EPA changed approximately 2.3 fold from baseline; DHA did not change from baseline in INT group; no changes in control group	**TBARS:** Significant increase (INT at 48 h & 72 h post damage) **All other outcomes measures:** No significant change	−ve (TBARS only)
Lembke et al. (2014) [42]	Untrained Tier 0 Males/Females (*n* = 64) **Age (Mean ± SD)**: INT: 18.6 ± 1.2; PLA: 18.9 ± 1.1 years **INT**: *n* = 42 **PLA**: *n* = 22	**Mass of oil (mg)**: No data **EPA + DHA + DPA (mg)**: 2700 **Brand**: KD Pharma, Bexbach, Germany **Placebo**: Sunflower oil **Supplement period (days)**: 30	No data	Multiple sets of maximum eccentric forearm extensions performed with the non-dominant arm; each group did two sets of 30 reps	DOMS ROM CK CRP	Moderate pain	No data	**DOMS:** Significant reduction (INT at 72 h & 96 h post damage) **CRP:** Significant reduction (INT at 24 h post damage) **All other outcomes measures:** No significant change	+ve (DOMS, CRP)
Marques et al. (2015) [43]	Wheelchair basketball players Tier 3 Males (*n* = 8) **Age (Mean ± SD)**: 33.8 ± 8.3 years **INT**: *n* = 8 **PLA**: *n* = 0	**Mass of oil (mg)**: 3000 **EPA + DHA + DPA (mg)**: 1800 **Brand**: Naturalis, Brazil **Placebo**: Not placebo controlled **Supplement period (days)**: 30	No data	Training performed 4 times/week for 4 h/session; the training intensity of the acute exercise was estimated according to the peak heart rate and average heart rate during 60 min of basketball play	CK LDH CRP IL-6 IL-1ra TNF-α IL-8 IL-1b IL-4	No data	No data	**LDH, IL-1ra, and IL-6:** Significant reduction in INT **IL-8:** Significant increase in INT **All other outcomes measures:** No significant change	+ve (LDH, IL-1ra, IL-6) −ve (IL-8)
Mickleborough et al. (2015) [44]	Untrained Tier 0 Males (*n* = 32) **Age (Mean ± SD)**: 22.0 ± 2 years **INT**: *n* = 16 **PLA**: *n* = 16	**Mass of oil (mg)**: 1200 **EPA + DHA + DPA (mg)**: 400 **Brand**: Pharmalink International Ltd., Hong Kong **Placebo**: Olive oil **Supplement period (days)**: 30	Pill counting	20 min downhill running at −16% gradient and 70% of VO2max	DOMS MVC ROM Mb TNF-α CK	Mild pain	No data	**Mb and TNF-α:** Significant reduction (INT at 24 h, 48 h, 72 h, & 96 h post damage) **DOMS:** Significant reduction (INT at 72 h & 96 h post damage) **ROM:** Significant increase (INT at 96 h post damage) **All other outcomes measures:** No significant change	+ve (Mb, TNF-α, ROM, DOMS)
Corder et al. (2016) [45]	Untrained Tier 0 Females (*n* = 27) **Age (Mean ± SD)**: 33 ± 2 years **INT**: *n* = 14 **PLA**: *n* = 13	**Mass of oil (mg)**: No data **EPA + DHA + DPA (mg)**: 3000 **Brand**: DSM Nutritional Products, USA **Placebo**: Corn oil & soy oil without *n*-3 **Supplement period (days)**: 9	Pill counting	4 sets of eccentric bicep curls to failure at 120% of 1RM; each eccentric phase lasted 4 s, with 3 min rest between sets	DOMS CRP UAC Stiffness	Moderate pain	No data	**DOMS:** Significant reduction (INT at 48 h post damage) **All other outcomes measures:** No significant change	+ve (DOMS only)
Tinsley et al. (2017) [46]	Untrained Tier 0 Females (*n* = 17) **Age (Mean ± SD)**: INT: 22.5 ± 1.8; PLA: 24.7 ± 1.6 **years** **INT**: *n* = 8 **PLA**: *n* = 9	**Mass of oil (mg)**: 6000 **EPA + DHA + DPA (mg)**: 3600 **Brand**: Cooper Institute, USA **Placebo**: Corn/soy oil **Supplement period (days)**: 14	Self-reported by supplement compliance form	10 sets to failure on elbow flexion and leg extension machines at 50% of the 1RM determined during the first visit; participants were instructed to keep a cadence of one second for the concentric portion of the movement and four seconds for the eccentric portion with 2 min rest between sets	DOMS	Severe pain	No data	**All outcomes measures:** No significant change	No effect
Tsuchiya et al. (2016) [47]	Untrained Tier 0 Males (*n* = 24) **Age (Mean ± SD)**:19.5 ± 0.8 years **INT**: *n* = 12 **PLA**: *n* = 12	**Mass of oil (mg)**: 2400 **EPA + DHA + DPA (mg)**: 860 **Brand**: Nippon Suisan Kaisha Ltd., Japan **Placebo**: Corn oil **Supplement period (days)**: 62	No data	5 sets of 6 maximal ECCs (elbow flexor contractions) of the bicep muscles −30 °/s from 90 deg to full extension; 3 s passive recovery between contractions	DOMS MVC ROM IL-6 Mb TNF-α CK UAC	Mild pain	Serum levels of DHA did not change but EPA increased in INT group; percentage not reported	**MVC:** Significant increase (INT at 24 h, 48 h, & 120 h post damage) **ROM:** Significant increase (INT immediately and at 24 h, 48 h, & 72 h post damage) **DOMS and IL-6:** Significant reduction (INT at 72 h post damage) **All other outcomes measures:** No significant change	+ve (MVC, ROM, DOMS, IL-6)
Jakeman et al. (2017) [48]	Recreationally active Tier 1 Males (*n* = 30) **Age (Mean ± SD)**: 26 ±4 years **INTs**: High EPA: *n* = 9; low EPA: *n* = 9 **PLA**: *n* = 9	**Mass of oil (mg)**: 8000 **EPA + DHA + DPA (mg)**: 6400, 2000 **Brand**: Take *n*-3 **Placebo**: Filler oil, flavor masker and gelatine **Supplement period** **(days)**: 0	No data	10 sets of 10 plyometric drop jumps	DOMS CK IL-6 Jump performance	Mild pain	No data	**Jump Performance:** Significant increase (INTs at 24 h, 48 h, 72 h, & 96 h post damage) **All other outcomes measures:** No significant change	+ve (Jump performance only)
McKinley-Barnard et al. (2018) [49]	Recreationally active Tier 1 Females (*n* = 22) **Age (Mean ± SD)**: 20.9 ± 1.4 years **INT**: *n* = 11 **PLA**: *n* = 11	**Mass of oil (mg)**: 6000 **EPA + DHA + DPA (mg)**: 4200 **Brand**: MusclePharm, USA **Placebo**: Safflower oil **Supplement period (days)**: 21	Self-reported by supplement compliance form	10 sets of 10 reps of knee extensors with 3 min of rest between sets at an isokinetic 10 speed of 30 °/s	DOMS SOD TNF-α Mb	Mild pain	No data	**DOMS:** Significant increase (INT post damage) **SOD:** Significant increase (INT post damage) **Mb:** significant increase (INT post damage) **All other outcomes measures:** No significant change	+ve (SOD only) −ve (DOMS, Mb)
Ochi et al. (2017) [50]	Untrained Tier 0 Males (*n* = 21) **Age (Mean ± SD)**: 21.0 ± 0.8 years **INT**: *n* = 10 **PLA**: *n* = 11	**Mass of oil (mg)**: 2400 **EPA + DHA + DPA (mg)**: 860 **Brand**: Nippon Suisan Kaisha Ltd., Japan **Placebo**: Corn oil **Supplement period (days)**: 62	Pill counting	6 sets of 10 maximal ECCs of elbow flexors with 2 min rest between sets; reps performed at 30 °/s	DOMS MVC ROM UAC	Moderate pain	Blood EPA increased significantly but DHA did not change in INT group; percentage not reported; no changes in control group	**DOMS:** Significant reduction (INT at 24 h & 48 h post damage) **MVC:** Significant increase (INT at 24 h post damage) **ROM:** Significant increase (INT immediately & at 48 h post damage) **All other outcomes measures:** No significant change	+ve (DOMS, MVC, ROM)
Black et al. (2018) [51]	Rugby union players Tier 4 Males (*n* = 20) **Age (Mean ± SD)**: 22.7± 2.11 years **INT**: *n* = 9 **PLA**: *n* = 11	**Mass of oil (mg)**: 1546 **EPA + DHA + DPA (mg)**: 1102 **Brand**: Smartfish, Germany **Placebo**: Same as supplement without fish oil **Supplement period** **(days)**: 35	Self-reported	Training 5 days per week: sessions included strength and conditioning, match skills/simulated match play and flexibility on 4 days; one recovery day consisted of light training	DOMS Jump performance	No data	Blood PUFA concentration increased by 2.69% in INT group; no changes in control group	**DOMS and Jump performance:** Significant reduction (INT following post supplementation)	+ve (DOMS only) −ve (Jump performance)
Philpott et al. (2018) [52]	Soccer players Tier 2 Males (*n* = 30) **Age (Mean ± SD)**: 23 ±1 years **INT**: *n* = 10 **PLAs**: Protein beverage: *n* = 10; CHO beverage: *n* = 10	**Mass of oil (mg)**: No data **EPA + DHA + DPA (mg)**: 2200 **Brand**: Smartfish Sports Nutrition Ltd. **Placebo**: Protein beverage, CHO beverage **Supplement period (days)**: 42	Blood sample	3 sets of 30 reps of knee flexion/extension with 1 min rest between sets-hamstrings	DOMS MVC CK CRP	Severe pain	Blood LC *n*-3 PUFAs/total PUFAs composition increased by 58% from baseline in INT group; no changes in control groups	**DOMS:** Significant reduction (INT at 24 h, 48 h & 72 h post damage) **CK:** Significant reduction (INT at 72 h post damage) **All other outcomes measures:** No significant change	+ve (DOMS, CK)
Tsuchiya et al. (2019) [53]	Untrained Tier 0 Males (*n* = 16) **Age (Mean ± SD)**: INT: 20.9 ± 0.4; PLA: 21.9 ± 1.4 years **INT**: *n* = 8 **PLA**: *n* = 8	**Mass of oil (mg)**: 2400 **EPA + DHA + DPA (mg)**: 860 **Brand**: Nippon Suisan Kaisha Ltd., Japan **Placebo**: Corn oil **Supplement period (days)**: 62	Pill counting	6 sets of 10 maximal voluntary ECCs of elbow flexors with a rest period of 90 s between each set	DOMS MVC ROM UAC Stiffness	Moderate pain	Serum levels of EPA and DHA significantly increased in INT group; percentage not reported	**MVC:** Significant increase (INT immediately & at 24 h post damage) **ROM:** Significant increase (INT immediately and at 24 h, 48 h, & 120 h post damage) **DOMS:** Significant reduction (INT at 120 h post damage) **UAC:** Significant reduction (INT at 48 h & 120 h post damage) **Stiffness:** Significant reduction (INT immediately & at 48 h post damage)	+ve (MVC, ROM, DOMS, UAC, Stiffness)
Barenie et al. (2022) [54]	Untrained Tier 0 Males (*n* = 49) **Age (Mean ± SD)**: 21.7 ± 2.4 years **INTs**: ESPO572: *n* = 26; PSCO524: *n* = 23 **PLA**: *n* = 16 (from previous work Mickleborough 2015 [44])	**Mass of oil (mg)**: PCSO524 = 200; ESPO572 = 200 **EPA + DHA + DPA (mg)**: PCSO524 = 64; ESPO572 = 18.8 **Brand**: Pharma link International Ltd., Hong Kong **Placebo**: Olive oil **Supplement period** **(days)**: 29	Blood sample	Downhill running speed eliciting 70% of heart rate at VO2 peak for 20 min at16% gradient	DOMS MVC ROM CK IL-6 TNF-α Peak power	Mild pain	No significant change in O3I	**DOMS, CK, and TNF-α**: Significant reduction (INTs at 24 h, 48 h & 72 h post damage) **ROM and MVC:** Significant increase (INTs at 24 h, 48 h & 72 h post damage)	+ve (DOMS, CK, TNF-α, ROM, MVC)
Buonocore et al. (2020) [55]	Untrained and trained runners Tier 0 Tier 2 Males/Females (*n* = 39) **Age (Mean ± SD)**: 23.80 ± 5.88 years **INTs**: *n* = 39 **PLA**: *n* = 0	**Mass of oil (mg)**: 3800 **EPA + DHA + DPA (mg)**: 2400 **Brand**: EthicSport, Italy **Placebo**: No data **Supplement period (days)**: 56	Pill counting	Participating in national and international running competitions vs performing physical activity no more than twice a week, for a maximum of one hour each time	CK SOD TNF-α LDH MDA GPx CAT	No data	No data	**TNF-α and MDA:** Significant reduction (trained group) **GPx and CAT:** Significant increase (trained & untrained groups) **All other outcomes measures:** No significant change	+ve (TNF-α, MDA, GPx, CAT)
Morishima et al. (2020) [56]	Untrained Tier 0 Males (*n* = 19) **Age (Mean ± SD)**: 20.8 ± 1.5 years **INT**: *n* = 10 **PLA**: *n* = 9	**Mass of oil (mg)**: 2400 **EPA + DHA + DPA (mg)**: 860 **Brand**: Nippon Suisan Kaisha Ltd., Japan **Placebo**: Corn oil **Supplement period (days)**: 57	Pill counting/blood sample	Knee extensor load with weights equal to 40% of body weight for 4 sets in total; resting between sets of 20, 30, and 40 s, respectively	MVC	No data	Serum EPA and DHA significantly increased in INT group; no changes in control group	**All outcomes measures:** No significant change	No effect
Ramos-Campo et al. (2020) [57]	Recreationally active/endurance trained Tier 1 Males (*n* = 15) **Age (Mean ± SD)**: 36.0 ± 8.1 years **INT**: *n* = 15 **PLA**: *n* = 15	**Mass of oil (mg)**: 3000 **EPA + DHA + DPA (mg)**: 2430 **Brand**: Brudy Plus, Brudytechnology, Spain **Placebo**: Olive oil **Supplement period (days)**: 70	Pill counting	8 sets of 6 reps of half-squats at 110% of 1RM with 2 min rest between sets	DOMS CK LDH IL1β IL-6 IL8 TNF-α CRP	Moderate pain	No data	**DOMS, LDH, and IL1β:** Significant reduction (INT immediately and at 24 h & 48 h post damage) **IL-6:** Significant reduction (INT immediately & at 24 h post damage) **CK:** Significant reduction (INT at 24 h post damage) **All other outcomes measures:** No significant change	+ve (DOMS, CK, LDH, IL1β, IL-6)
VanDusseldorp et al. (2020) [58]	Recreationally active/strength trained Tier 1 Males/Females (*n* = 32) **Age (Mean ± SD)**: M: 23.8 ± 2.7; F: 23.4 ± 3.1 years **INTs**: 6 g: *n* = 8; 4 g: *n* = 8; 2 g: *n* = 8 **PLA**: *n* = 8	**Mass of oil (mg)**: 6000, 4000, 2000 **EPA + DHA + DPA (mg)**: 4200, 2800, 1400 **Brand**: Muscle Pharm, USA **Placebo**: Safflower oil **Supplement period** **(days)**: 52	Pill counting	10 sets of 8 reps of eccentric squats (4 s lowering phase and 1 s upward phase) at 70% of 1RM with 3 min rest between sets; then, 5 sets of 20 split squat jumps with 2 min rest between sets	DOMS MVC VJ CK LDH	Severe-to-moderate pain	No data	**DOMS, MVC, CK, and LDH:** Significant reduction (INTs at 24 h, 48 h & 72 h post damage) **All other outcomes measures:** No significant change	+ve (DOMS, CK, LDH) −ve (MVC)
Kyriakidou et al. (2021) [59]	Recreationally active Tier 1 Males (*n* = 14) **Age (Mean ± SD)**: 24.5 ± 3.9 years **INT**: *n* = 7 **PLA**: *n* = 7	**Mass of oil (mg)**: 3900 **EPA + DHA + DPA (mg)**: 3003 **Brand**: Natures Best, UK **Placebo**: Collagen **Supplement period** **(days)**: 28	Pill counting	Downhill running-60 min, 65% VO2max, 10% gradient	DOMS MVC CK IL-6 TNF-α Peak power	Moderate pain	No data	**DOMS:** Significant reduction (INT at 24 h post damage) **All other outcomes measures:** No significant change	+ve (DOMS only)
Loss et al. (2022) [60]	Recreationally active Tier 1 Females (*n* = 30) **Age (Mean ± SD)**: 22.2 ± 3.3 years **INT**: *n* = 15 **PLA**: *n* = 15	**Mass of oil (mg)**: No data **EPA + DHA + DPA (mg)**: 3200 **Brand**: Vital Atman, Brazil **Placebo**: Olive oil **Supplement period (days)**: 4	Self-reported by food record form	10 sets of 10 unilateral eccentric repetitions at 100% of 1RM test with 1 min rest between sets; keep a cadence of one second during the concentric phase of the movement (performed with both legs) and four seconds during the eccentric portion (performed only with the right leg)	DOMS MVC	Mild pain	No data	**All outcomes measures:** No significant change	No effect
Tsuchiya et al. (2021) [61]	Untrained Tier 0 Males (*n* = 22) **Age (Mean ± SD)**: INT: 20.4 ± 0.4; PLA: 19.8 ± 1.5 years **INT**: *n* = 11 **PLA**: *n* = 11	**Mass of oil (mg)**: 2400 **EPA + DHA + DPA (mg)**: 860 **Brand**: Nippon Suisan Kaisha Ltd., Japan **Placebo**: Corn oil **Supplement period (days)**: 33	Pill counting	6 sets of 10 maximal voluntary ECCs of elbow flexors with a rest period of 90 s between each set	DOMS MVC ROM UAC IL-6 CK Thickness	Moderate pain	Blood EPA concentration increased, with no significant change in DHA in INT group; percentage not reported	**ROM:** Significant increase (INT immediately post damage) **CK:** Significant reduction (INT at 72 h post damage) **All other outcomes measures:** No significant change	+ve (ROM, CK)
Visconti et al. (2021) [62]	Resistance trained Tier 1 Males (*n* = 26) **Age (Mean ± SD)**: 23 ± 4 years **INTs**: 8 g: *n* = 7; 6 g: *n* = 10 **PLA**: *n* = 9	**Mass of oil (mg)**: 6000, 8000 **EPA + DHA + DPA (mg)**: 1800, 2400 **Brand**: Beast Sports Nutrition, USA **Placebo**: CLA **Supplement period (days)**: 33	Self-reported	10 sets of 8 barbell back squats at 70% 1RM with 3 min rest between sets, followed by split squat jumps	DOMS ROM VJH CK	Moderate pain	No data	**All outcomes measures:** No significant change	No effect
Ayubi et al. (2022) [63]	Untrained Tier 0 Males (*n* = 20) **Age (Mean ± SD)**: INT: 27.30 ± 8.21; PLA: 23.10 ± 6.13 years **INT**: *n* = 10 **PLA**: *n* = 10	**Mass of oil (mg)**: 1000 **EPA + DHA + DPA (mg)**: 900 **Brand**: No data **Placebo**: No data **Supplement period (days)**: 1	No data	High-intensity weight training	DOMS TNF-α	Moderate pain	No data	**DOMS and TNF-α:** Significant reduction (INT post damage)	+ve (DOMS, TNF-α)
Asjodi et al. (2023) [64]	Untrained Tier 0 Males (*n* = 48) **Age (Mean ± SD)**: INT: 22.16 ± 2.28; PLA: 22.41 ± 1.88 years **INT**: *n* = 12 **PLA**: *n* = 12	**Mass of oil (mg)**: No data **EPA + DHA + DPA (mg)**: 1500 **Brand**: Karen Pharmaceutical Co, Iran **Placebo**: Maltodextrin **Supplement period (days)**: 28	No data	3 sets of 15 repetitions of eccentric knee extensions at 70% 1RM	CK LDH DOMS	No data	No data	**CK:** Significant reduction (INT at 48 h post damage) **LDH:** Significant reduction (INT at 24 h post damage) **DOMS:** Significant reduction (INT at 24 h & 48 h post damage)	+ve (CK, LDH, DOMS)
Barquilha et al. (2023) [65]	Untrained Tier 0 Males (*n* = 16) **Age (Mean ± SD)**: No data **INT**: *n* = 8 **PLA**: *n* = 8	**Mass of oil (mg)**: No data **EPA + DHA + DPA (mg)**: 1386 **Brand**: Naturalis Nutricao & Farma LTDA, Brazil **Placebo**: No data **Supplement period (days)**: 42	Pill counting	Strength training protocol: weeks 1, 3, and 6 (hypertrophy)—6 series of 10 repetitions with a 1 min interval (6 × 10 with 1 min interval); weeks 2 and 4 (strength)—5 × 5 with a 3 min interval; week 5 (resistance)—2 × 20 with a 1 min interval	CK LDH CRP IL-6 IL-1b TNF-α	No data	No data	**CK, LDH, CRP, and IL-6:** Significant reduction (INT immediately and at 24 h & 48 h post damage) **All other outcomes measures:** No significant change	+ve (CK, LDH, CRP, IL-6)
Mackay et al. (2023) [66]	Recreationally active Tier 1 Males (*n* = 16) **Age (Mean ± SD)**: INT: 19.3 ± 1.5; PLA: 21.3 ± 2.7 years **INT**: *n* = 8 **PLA**: *n* = 8	**Mass of oil (mg)**: 5000 **EPA + DHA + DPA (mg)**: 2367 **Brand**: Select Supplement Inc. **Placebo**: Soybean **Supplement period (days)**: 32	Pill counting/blood sample	12 sets of isokinetic knee extensions and 12 sets of isokinetic knee flexions with the non-dominant leg; minimum of 60 s rest between sets; each set consisted of a pre-set workload based on 120% of peak isokinetic torque performed 10 times/set for 12 sets	DOMS CK Peak torque	Moderate pain	Blood LC *n*-3 PUFAs/total PUFAs increased by 14.9 percentage points in INT group; no changes in control group	**All outcomes measures:** No significant change	No effect
Yang et al. (2023) [67]	Recreationally active/resistance trained Tier 1 Males (*n* = 30) **Age (Mean ± SD)**: 20.4 ± 0.92 years **INT**: *n* = 15 **PLA**: *n* = 15	**Mass of oil (mg)**: 3000 **EPA + DHA + DPA (mg)**: 570 **Brand**: Aker Marine biology, SUPERBA, Norway **Placebo**: Soybean oil **Supplement period (days)**: 6	No data	10 sets of 8 repetitions of eccentric squats (3 s lowering phase and 1 s upward phase) at 70% of 1RM with 3 min of rest between sets; after completing, participants performed 5 sets of 20 consecutive bodyweight split jump squats, resting 3 min between sets	CK LDH SOD MDA IL-2 IL-6 TNF-α T-AOC Peak torque	No data	No data	**CK:** Significant reduction (INT at 24 h & 48 h post damage) **MDA:** Significant reduction (INT at 6 h post damage) **SOD:** Significant increase (INT immediately and at 6 h & 24 h post damage) **T-AOC:** Significant increase (INT immediately and at 6 h & 72 h post damage) **Peak torque:** Significant increase (INT at 24 h & 48 h post damage) **All other outcomes measures:** No significant change	+ve (CK, MDA, SOD, T-AOC, Peak torque)
Heileson et al. (2024) [68]	Recreationally active Tier 1 Males (*n* = 30) **Age (Mean ± SD)**: INTs: EPA + DHA: 20.5 ± 2.6; EPA: 22.6 ± 4.7; DHA: 19.1 ± 1.2 PLA: 24.1 ± 7.0 years **INTs**: EPA + DHA: *n* = 8; EPA: *n* = 8; DHA: *n* = 7 **PLA**: *n* = 7	**Mass of oil (mg)**: No data **EPA + DHA + DPA (mg)**: 4000 **Brand**: Carlson Labs, Arlington Heights, USA **Placebo**: Coconut oil **Supplement period (days)**: 52	Pill counting/blood sample	Two separate protocols: downhill at a grade of 16%, 20 min at 70% VO2max; followed by resting for 2 min, then the plyometric section consisted of 5 sets of 20 jumping lunges with a 2 min rest between each set	DOMS ROM CK CRP Jump performance Peak power Lower body strength	Moderate pain	O3I significantly increased in all INT groups; no changes in control group	**DOMS:** Significant reduction (INTs at 48 h post damage) **Jump performance:** Significant increase (INTs at 1 h & 48 h post damage) **Lower body strength:** Significant increase (INTs at 24 h & 72 h post damage) **Peak power:** Significant increase (INTs at 48 h post damage) **All other outcomes measures:** No significant change	+ve (DOMS, Jump performance, Strength, Peak power)
Posnakidis et al. (2024) [69]	Untrained Tier 0 Males/Females (*n* = 19) **Age (Mean ± SD)**: INT: 29 ± 6; PLA: 30 ± 3 years **INT**: *n* = 10 **PLA**: *n* = 9	**Mass of oil (mg)**: No data **EPA + DHA (mg)**: 6300 **Brand**: Palupa Medical, Nicosia, Cyprus **Placebo**: Extra virgin olive oil **Supplement period (days)**: 56	Blood sample	High-intensity functional training included squats, medicine ball crunches (3–4 kg), clean and presses, box jumps, TRX chest presses, wall ball throws, burpees, sledgehammers, and 10 m sprints, at 60% 1RM for weight-bearing exercises	VJ CK CRP	No data	No data	**All outcomes measures:** No significant change	No effect
Makaje et al. (2024) [70]	Untrained Tier 0 Males (*n* = 24) **Age (Mean ± SD)**: INT: 21.17 ± 3.33; PLA: 21.17 ± 4.17 years **INT**: *n* = 12 **PLA**: *n* = 12	**Mass of oil (mg)**: 4000 **EPA + DHA (mg)**: 2800 **Brand** Pronova Pure, Newtrition, BASF, Singapore **Placebo**: Soybean oil **Supplement period (days)**: 30	Pill counting/blood sample	High-intensity interval training cycling sessions consisted of 8 s of high-intensity cycling followed by 12 s of slow cycling continuously throughout a 20 min session	DOMS CK CRP	No data	O3I significantly increased in INT group (52.51%); no changes in control group	**DOMS:** Significant reduction (INT post damage) **CK:** Significant reduction (INT at 48 h post damage) **All other outcomes measures:** No significant change	+ve (DOMS, CK)

**Table 2:** Summary of EPA and DHA supplement types in the included studies (*n* = 43) presented in Table 1.

Supplement Type	Number of Studies (%)
**EPA + DHA combination**	**41 (95%)**
EPA dominant	32 (78%)
DHA dominant	4 (9.7%)
Equal ratio	2 (4.8%)
Unspecified	3 (7.3%)
**Isolated EPA**	
Unspecified	1 (2.3%)
**Isolated DHA**	
Methyl ester	1 (2.3%)

**Table 3:** Summary of administration methods, sources, brand reporting, and placebo types in the included studies (*n* = 43) presented in Table 1.

Category	Number of Studies (%)
**Administration methods**	
Capsule	36 (83.7%)
Beverage	3 (7.0%)
Not specified	4 (9.3%)
**Sources of LC *n*-3 PUFAs**	
Fish derived	26 (60.4%)
Algae oil	1 (2.3%)
**Krill oil**	1 (2.3%)
Green-lipped mussel	1 (2.3%)
Green-lipped mussel + krill oil	1 (2.3%)
Not specified	13 (30.2%)
**Brand reporting**	
Brand specified	41 (95%)
**Placebo types**	
Reported	35 (81.4%)
Oil-based placebo (within reported)	23 (65.7%)
Not specified	8 (18.6%)

**Table 4:** Summary of biological assessments of LC *n*-3 PUFA status and compliance methods in the included studies (*n* = 43) presented in Table 1.

Assessment Type	Number of Studies (%)
**Biological assessment**	(% of 18)
Whole blood (venous)	7 (38.8%)
Plasma	2 (11.1%)
Serum	5 (27.7%)
Neutrophil membranes	1 (5.5%)
Direct assessment of O3I	1 (5.5%)
Indirect assessment of O3I	2 (11.1%)
**Compliance assessment**	(% of 27)
At least one method reported	27 (100%)
Pill counting	15 (55.6%)
Self-reporting only	6 (22.2%)
Other/not specified	6 (22.2%)

**Table 5:** Summary of gender distribution and power calculation reporting in the included studies (*n* = 43) presented in Table 1.

Category	Number of Studies (%)
**Gender distribution**	
Male only	31 (72.0%)
Female only	5 (11.6%)
Both male and female	7 (16.3%)
**Power calculation**	
Reported	15 (34.8%)
Not reported	28 (65.2%)

**Table 6:** Summary of exercise models used to induce muscle damage in the included studies (*n* = 43) presented in Table 1.

Category	Number of Studies (%)
**Non-EIMD studies**	**3 (7%)**
**EIMD studies**	
**Resistance exercise**	**29 (72.5%)**
Eccentric only, machine based	11 (38% resistance)
Eccentric only, no machines	4 (14% resistance)
Combined eccentric + concentric	12 (41% resistance)
Not specified	2 (7% resistance)
**Endurance exercise**	**9 studies (23%)**
**Combined endurance + resistance**	**2 studies (5%)**
**Muscle groups (resistance)**	
Lower body (hamstrings/quadriceps)	15 (53.5%)
Upper body (elbow flexors)	10 (37.5%)
Both upper and lower	1 (3.5%)
Not specified	2 (7%)

**Table 7:** Summary of DOMS and muscle damage biomarker assessments following LC *n*-3 PUFA supplementation in the included studies (*n* = 43) presented in Table 1.

Category	Number of Studies (%)
**DOMS**	
Reported	32 (74.4%)
**Assessment methods**	
100 mm VAS	27 (62.7%)
Other scales	5 (11.6%)
**Effect of LC *n*-3 PUFAs**	
Reduced DOMS severity	19 (59.0% of DOMS studies)
Increased DOMS severity	1 (3.0% of DOMS studies)
No effect	12 (37.5% of DOMS studies)
**Muscle damage biomarkers**	
Reported	32 (74.4%)
**Markers measured**	
CK only	18 (56.2% of biomarker studies)
Mb only	1 (3.1% of biomarker studies)
CK + LDH + Mb	13 (40.6% of biomarker studies)
**Effect of LC *n*-3 PUFAs**	
Reduced biomarkers	15 (46.8% of biomarker studies)
Increased biomarkers	1 (3.1% of biomarker studies)
No effect	16 (50.0% of biomarker studies)

**Table 8:** Summary of muscle function assessments following LC *n*-3 PUFA supplementation in the included studies (*n* = 43) presented in Table 1.

Category	Number of Studies (%)
**ROM**	
Reported	14 (56%)
**Effect of LC *n*-3 PUFAs**	
Improved ROM	7 (50% of ROM studies)
Reduced ROM	4 (28% of ROM studies)
No effect	3 (21% of ROM studies)
**MVC**	
Reported	13 (52%)
**Effect of LC *n*-3 PUFAs**	
Improved MVC	5 (38% of MVC studies)
Reduced MVC	1 (8% of MVC studies)
No effect	7 (54% of MVC studies)
**Jump performance (JP)**	
Reported	6 (24%)
**Effect of LC *n*-3 PUFAs**	
Improved JP	2 (33% of JP studies)
Reduced JP	1 (17% of JP studies)
No effect	3 (50% of JP studies)
**Peak power**	
Reported	3 (12%)
**Effect of LC *n*-3 PUFAs**	
Improved peak power	1 (33% of peak power studies)
No effect	2 (67% of peak power studies)
**Peak torque**	
Reported	2 (8%)
**Effect of LC *n*-3 PUFAs**	
Improved peak torque	1 (50% of peak torque studies)
No effect	1 (50% of peak torque studies)

**Table 9:** Summary of inflammatory marker assessments following LC *n*-3 PUFA supplementation in the included studies (*n* = 43) presented in Table 1.

Category	Number of Studies (%)
**IL-6**	
Reported	15 (55.5%)
**Effect of LC *n*-3 PUFAs**	
Reduced IL-6	5 (33.3% of IL-6 studies)
Increased IL-6	1 (6.7% of IL-6 studies)
No effect	9 (60% of IL-6 studies)
**TNF-α**	
Reported	15 (55.5%)
**Effect of LC *n*-3 PUFAs**	
Reduced TNF-α	6 (40% of TNF-α studies)
No effect	9 (60% of TNF-α studies)
**CRP**	
Reported	14 (51.8%)
**Effect of LC *n*-3 PUFAs**	
Reduced CRP	5 (35.7% of CRP studies)
No effect	9 (64.2% of CRP studies)
**IL-1ra**	
Reported	4 (14.8%)
**Effect of LC *n*-3 PUFAs**	
Reduced IL-1ra	1 (25% of IL-1ra studies)
No effect	3 (75% of IL-1ra studies)
**IL-1β**	
Reported	3 (11%)
**Effect of LC *n*-3 PUFAs**	
Reduced IL-1β	1 (33.3% of IL-1β studies)
No effect	2 (66.7% of IL-1β studies)
**IL-8**	
Reported	3 (11%)
**Effect of LC *n*-3 PUFAs**	
Increased IL-8	1 (33.3% of IL-8 studies)
No effect	2 (66.7% of IL-8 studies)
**IL-2**	
Reported	1 (3.7%)
**Effect of LC *n*-3 PUFAs**	
No effect	1 (100% of IL-2 studies)
**IL-4**	
Reported	1 (3.7%)
**Effect of LC *n*-3 PUFAs**	
No effect	1 (100% of IL-4 studies)

**Table 10:** Summary of oxidative stress marker assessments following LC *n*-3 PUFA supplementation in the included studies (*n* = 43) presented in Table 1.

Category	Number of Studies (%)
**MDA**	
Reported	6 (75%)
**Effect of LC *n*-3 PUFAs**	
Reduced MDA	3 (50% of MDA studies)
No effect	3 (50% of MDA studies)
**SOD**	
Reported	4 (50%)
**Effect of LC *n*-3 PUFAs**	
Increased SOD	3 (75% of SOD studies)
No effect	1 (25% of SOD studies)
**CAT**	
Reported	2 (25%)
**Effect of LC *n*-3 PUFAs**	
Increased CAT	2 (100% of CAT studies)
**GPx**	
Reported	2 (25%)
**Effect of LC *n*-3 PUFAs**	
Increased GPx	1 (50% of GPx studies)
No effect	1 (50% of GPx studies)
**T-AOC**	
Reported	1 (12.5%)
**Effect of LC *n*-3 PUFAs**	
Increased T-AOC	1 (100% of T-AOC studies)
**TBARS**	
Reported	1 (3.7%)
**Effect of LC *n*-3 PUFAs**	
Increased TBARS	1 (100% of TBARS studies)

**Table 11:** Summary of direct muscle swelling assessments following LC *n*-3 PUFA supplementation in the included studies (*n* = 43) presented in Table 1.

Category	Number of Studies (%)
**UAC**	
Reported	7 (23%)
**Effect of LC *n*-3 PUFAs**	
Reduced UAC	1 (14.2% of UAC studies)
No effect	6 (86% of UAC studies)
**Muscle Stiffness**	
Reported	2 (6.7%)
**Effect of LC *n*-3 PUFAs**	
Reduced Stiffness	1 (50% of stiffness studies)
No effect	1 (50% of stiffness studies)
**Muscle Thickness**	
Reported	1 (3.3%)
**Effect of LC *n*-3 PUFAs**	
No effect	1 (100% of thickness studies)

## Data Availability

Extracted data from the meta-analysis can be made available upon reasonable request to the corresponding author.

## Supplementary Materials

The following supporting information can be downloaded at: https://www.mdpi.com/article/10.3390/nu18091447/s1, Figure S1: Risk of bias: authors’ judgments regarding each risk of bias item for included studies using Cochrane Risk of Bias tool for randomized trials; Figure S2: The most common problems found in the risk of bias in selected studies; Figure S3: Methodological quality assessment for included studies: McMaster Critical Review Form for Quantitative Studies; Figure S4: Methodological quality according to the PEDro Scale; Figure S5: Funnel plots of delayed onset muscle soreness (DOMS), creatine kinase (CK), muscle strength, range of motion (ROM), and muscle swelling; Table S1: Leave-one-out meta-analysis (DOMS outcome); Table S2: Leave-one-out meta-analysis (CK outcome); Table S3: Leave-one-out meta-analysis (maximum strength outcome); Table S4: Leave-one-out meta-analysis (ROM outcome); Table S5: Leave-one-out meta-analysis (swelling outcome).

## Abbreviations

BMI	Body mass index
CAT	Catalase
CK	Creatine kinase
CON	Control
CRP	C-reactive protein
DHA	Docosahexaenoic acid
DOMS	Delayed onset muscle soreness
DPA	Docosapentaenoic acid
ECC	Eccentric
EIMD	Exercise-induced muscle damage
EPA	Eicosapentaenoic acid
GPx	Glutathione peroxidase
IL-1ra	Interleukin-1 receptor antagonist
IL-1β	Interleukin-1β
IL-2	Interleukin-2
IL-4	Interleukin-4
IL-6	Interleukin-6
IL-8	Interleukin-8
INT	Intervention
LC *n*-3 PUFA	Long-chain omega-3 polyunsaturated fatty acid
LDH	Lactate dehydrogenase
Mb	Myoglobin
MDA	Malondialdehyde
MDS	Muscle damage stimulus
MVC	Maximal voluntary contraction
NSAIDs	Non-steroidal anti-inflammatory drugs
O3I	Omega-3 index
PLA	Placebo
RBC	Red blood cell
RCT	Randomized controlled trial
ROM	Range of motion
SOD	Superoxide dismutase
T-AOC	Total antioxidant capacity
TBARS	Thiobarbituric acid reactive substances
TNF-α	Tumor necrosis factor-α
UAC	Upper arm circumference
VAS	Visual analog scale

## References

[1] Owens D.J., Twist C., Cobley J.N., Howatson G., Close G.L. (2019). Exercise-induced muscle damage: What is it, what causes it and what are the nutritional solutions?. Eur. J. Sport Sci..

[2] McKune A., Semple S., Peters-Futre E. (2012). Acute exercise-induced muscle injury. Biol. Sport.

[3] Warren G.L., Lowe D.A., Armstrong R.B. (1999). Measurement Tools Used in the Study of Eccentric Contraction???Induced Injury. Sports Med..

[4] Guilhem G., Cornu C., Guével A. (2010). Neuromuscular and muscle-tendon system adaptations to isotonic and isokinetic eccentric exercise. Ann. Phys. Rehabil. Med..

[5] Peake J.M., Neubauer O., Della Gatta P.A., Nosaka K. (2017). Muscle damage and inflammation during recovery from exercise. J. Appl. Physiol..

[6] Langston P.K., Mathis D. (2024). Immunological regulation of skeletal muscle adaptation to exercise. Cell Metab..

[7] Cheung K., Hume P., Maxwell L. (2003). Delayed onset muscle soreness: Treatment strategies and performance factors. Sports Med..

[8] Gulick D.T., Kimura I.F. (1996). Delayed Onset Muscle Soreness: What Is It and How Do We Treat It?. J. Sport Rehabil..

[9] Lilja M., Mandić M., Apró W., Melin M., Olsson K., Rosenborg S., Gustafsson T., Lundberg T.R. (2017). High doses of anti-inflammatory drugs compromise muscle strength and hypertrophic adaptations to resistance training in young adults. Acta Physiol..

[10] Lundberg T.R., Howatson G. (2018). Analgesic and anti-inflammatory drugs in sports: Implications for exercise performance and training adaptations. Scand. J. Med. Sci. Sports.

[11] McGlory C., Galloway S.D., Hamilton D.L., McClintock C., Breen L., Dick J.R., Bell J.G., Tipton K.D. (2014). Temporal changes in human skeletal muscle and blood lipid composition with fish oil supplementation. Prostaglandins Leukot. Essent. Fat. Acids.

[12] Naderi A., Rothschild J.A., Santos H.O., Hamidvand A., Koozehchian M.S., Ghazzagh A., Berjisian E., Podlogar T. (2025). Nutritional Strategies to Improve Post-exercise Recovery and Subsequent Exercise Performance: A Narrative Review. Sports Med..

[13] Ruggiero M., Motti M.L., Meccariello R., Mazzeo F. (2025). Resveratrol and Physical Activity: A Successful Combination for the Maintenance of Health and Wellbeing?. Nutrients.

[14] Kapoor B., Kapoor D., Gautam S., Singh R., Bhardwaj S. (2021). Dietary Polyunsaturated Fatty Acids (PUFAs): Uses and Potential Health Benefits. Curr. Nutr. Rep..

[15] Goldberg R.J., Katz J. (2007). A meta-analysis of the analgesic effects of omega-3 polyunsaturated fatty acid supplementation for inflammatory joint pain. Pain.

[16] Callan N., Hanes D., Bradley R. (2020). Early evidence of efficacy for orally administered SPM-enriched marine lipid fraction on quality of life and pain in a sample of adults with chronic pain. J. Transl. Med..

[17] Ko G.D., Nowacki N.B., Arseneau L., Eitel M., Hum A. (2010). Omega-3 fatty acids for neuropathic pain: Case series. Clin. J. Pain.

[18] Philpott J.D., Witard O.C., Galloway S.D. (2018). Applications of omega-3 polyunsaturated fatty acid supplementation for sport performance. Res. Sports Med..

[19] Calder P.C. (2017). Omega-3 fatty acids and inflammatory processes: From molecules to man. Biochem. Soc. Trans..

[20] Fernández-Lázaro D., Arribalzaga S., Gutiérrez-Abejón E., Azarbayjani M.A., Mielgo-Ayuso J., Roche E. (2024). Omega-3 Fatty Acid Supplementation on Post-Exercise Inflammation, Muscle Damage, Oxidative Response, and Sports Performance in Physically Healthy Adults—A Systematic Review of Randomized Controlled Trials. Nutrients.

[21] Heileson J.L., Funderburk L.K. (2020). The effect of fish oil supplementation on the promotion and preservation of lean body mass, strength, and recovery from physiological stress in young, healthy adults: A systematic review. Nutr. Rev..

[22] Lv Z.-T., Zhang J.-M., Zhu W.-T. (2020). Omega-3 Polyunsaturated Fatty Acid Supplementation for Reducing Muscle Soreness after Eccentric Exercise: A Systematic Review and Meta-Analysis of Randomized Controlled Trials. BioMed Res. Int..

[23] Xin G., Eshaghi H. (2021). Effect of omega-3 fatty acids supplementation on indirect blood markers of exercise-induced muscle damage: Systematic review and meta-analysis of randomized controlled trials. Food Sci. Nutr..

[24] Cumpston M., Li T., Page M., Chandler J., Welch V., Higgins J.P., Thomas J. (2019). Updated guidance for trusted systematic reviews: A new edition of the Cochrane Handbook for Systematic Reviews of Interventions. Cochrane Database Syst. Rev..

[25] Law M., Stewart D., Letts L., Pollock N., Bosch J., Westmorland M. (1998). Guidelines for Critical Review of Qualitative Studies.

[26] Moseley A.M., Elkins M.R., Van der Wees P.J., Pinheiro M.B. (2020). Using research to guide practice: The Physiotherapy Evidence Database (PEDro). Braz. J. Phys. Ther..

[27] McKay A.K.A., Stellingwerff T., Smith E.S., Martin D.T., Mujika I., Goosey-Tolfrey V.L., Sheppard J., Burke L.M. (2022). Defining Training and Performance Caliber: A Participant Classification Framework. Int. J. Sports Physiol. Perform..

[28] Toft A.D., Thorn M., Ostrowski K., Asp S., Møller K., Iversen S., Hermann C., Søndergaard S.R., Pedersen B.K. (2000). N-3 polyunsaturated fatty acids do not affect cytokine response to strenuous exercise. J. Appl. Physiol..

[29] Lenn J., Uhl T., Mattacola C., Boissonneault G., Yates J., Ibrahim W., Bruckner G. (2002). The effects of fish oil and isoflavones on delayed onset muscle soreness. Med. Sci. Sports Exerc..

[30] Phillips T., Childs A.C., Dreon D.M., Phinney S., Leeuwenburgh C. (2003). A Dietary Supplement Attenuates IL-6 and CRP after Eccentric Exercise in Untrained Males. Med. Sci. Sports Exerc..

[31] Bloomer R.J., E Larson D., Fisher-Wellman K.H., Galpin A.J., Schilling B.K. (2009). Effect of eicosapentaenoic and docosahexaenoic acid on resting and exercise-induced inflammatory and oxidative stress biomarkers: A randomized, placebo controlled, cross-over study. Lipids Health Dis..

[32] Nieman D.C., Henson D.A., McAnulty S.R., Jin F., Maxwell K.R. (2009). n-3 Polyunsaturated Fatty Acids Do Not Alter Immune and Inflammation Measures in Endurance Athletes. Int. J. Sport Nutr. Exerc. Metab..

[33] Poprzecki S., Zajac A., Chalimoniuk M., Waskiewicz Z., Langfort J. (2009). Modification of blood antioxidant status and lipid profile in response to high-intensity endurance exercise after low doses of ω-3 polyunsaturated fatty acids supplementation in healthy volunteers. Int. J. Food Sci. Nutr..

[34] Tartibian B., Maleki B.H., Abbasi A. (2009). The Effects of Ingestion of Omega-3 Fatty Acids on Perceived Pain and External Symptoms of Delayed Onset Muscle Soreness in Untrained Men. Clin. J. Sport Med..

[35] Jouris K.B., McDaniel J.L., Weiss E.P. (2011). The Effect of Omega-3 Fatty Acid Supplementation on the Inflammatory Response to eccentric strength exercise. J. Sports Sci. Med..

[36] Tartibian B., Maleki B.H., Abbasi A. (2011). Omega-3 Fatty Acids Supplementation Attenuates Inflammatory Markers After Eccentric Exercise in Untrained Men. Clin. J. Sport Med..

[37] Houghton D., Onambele G.L. (2012). Can a standard dose of eicosapentaenoic acid (EPA) supplementation reduce the symptoms of delayed onset of muscle soreness?. J. Int. Soc. Sports Nutr..

[38] Atashak S., Sharafi H., Azarbayjani M.A., Stannard S.R., Goli M.A., Haghighi M.M. (2013). Effect of omega-3 supplementation on the blood levels of oxidative stress, muscle damage and inflammation markers after acute resistance exercise in young athletes. Kinesiology.

[39] Rajabi A., Lotfi N., Abdolmaleki A., Rashid-Amiri S. (2013). The effects of omega-3 intake on delayed onset muscle sorness in non-athlet men. Pedagog. Psychol. Med.-Biol. Probl. Phys. Train..

[40] DiLorenzo F.M., Drager C.J., Rankin J.W. (2014). Docosahexaenoic Acid Affects Markers of Inflammation and Muscle Damage After Eccentric Exercise. J. Strength Cond. Res..

[41] Gray P., Chappell A., Jenkinson A.M., Thies F., Gray S.R. (2014). Fish Oil Supplementation Reduces Markers of Oxidative Stress But Not Muscle Soreness After Eccentric Exercise. Int. J. Sport Nutr. Exerc. Metab..

[42] Lembke P., Capodice J., Hebert K., Swenson T. (2014). Influence of omega-3 (n3) index on performance and wellbeing in young adults after heavy eccentric exercise. J. Sports Sci. Med..

[43] Marques C.G., Santos V.C., Levada-Pires A.C., Jacintho T.M., Gorjão R., Pithon-Curi T.C., Cury-Boaventura M.F. (2015). Effects of DHA-rich fish oil supplementation on the lipid profile, markers of muscle damage, and neutrophil function in wheelchair basketball athletes before and after acute exercise. Appl. Physiol. Nutr. Metab..

[44] Mickleborough T.D., A Sinex J., Platt D., Chapman R.F., Hirt M. (2015). The effects PCSO-524^®^, a patented marine oil lipid and omega-3 PUFA blend derived from the New Zealand green lipped mussel (*Perna canaliculus*), on indirect markers of muscle damage and inflammation after muscle damaging exercise in untrained men: A randomized, placebo controlled trial. J. Int. Soc. Sports Nutr..

[45] Corder K.E., Newsham K.R., McDaniel J.L., Ezekiel U.R., Weiss E.P. (2016). Effects of Short-Term Docosahexaenoic Acid Supplementation on Markers of Inflammation after Eccentric Strength Exercise in Women. J. Sports Sci. Med..

[46] Tinsley G.M., Gann J.J., Huber S.R., Andre T.L., La Bounty P.M., Bowden R.G., Gordon P.M., Grandjean P.W. (2016). Effects of Fish Oil Supplementation on Postresistance Exercise Muscle Soreness. J. Diet. Suppl..

[47] Tsuchiya Y., Yanagimoto K., Nakazato K., Hayamizu K., Ochi E. (2016). Eicosapentaenoic and docosahexaenoic acids-rich fish oil supplementation attenuates strength loss and limited joint range of motion after eccentric contractions: A randomized, double-blind, placebo-controlled, parallel-group trial. Eur. J. Appl. Physiol..

[48] Jakeman J.R., Lambrick D.M., Wooley B., Babraj J.A., Faulkner J.A. (2017). Effect of an acute dose of omega-3 fish oil following exercise-induced muscle damage. Eur. J. Appl. Physiol..

[49] McKinley-Barnard S.K., Andre T.L., Gann J.J., Hwang P.S., Willoughby D.S. (2018). Effectiveness of Fish Oil Supplementation in Attenuating Exercise-Induced Muscle Damage in Women During Midfollicular and Midluteal Menstrual Phases. J. Strength Cond. Res..

[50] Ochi E., Tsuchiya Y., Yanagimoto K. (2017). Effect of eicosapentaenoic acids-rich fish oil supplementation on motor nerve function after eccentric contractions. J. Int. Soc. Sports Nutr..

[51] Black K.E., Witard O.C., Baker D., Healey P., Lewis V., Tavares F., Christensen S., Pease T., Smith B. (2018). Adding omega-3 fatty acids to a protein-based supplement during pre-season training results in reduced muscle soreness and the better maintenance of explosive power in professional Rugby Union players. Eur. J. Sport Sci..

[52] Philpott J.D., Donnelly C., Walshe I.H., MacKinley E.E., Dick J., Galloway S.D., Tipton K.D., Witard O.C. (2018). Adding Fish Oil to Whey Protein, Leucine, and Carbohydrate Over a Six-Week Supplementation Period Attenuates Muscle Soreness Following Eccentric Exercise in Competitive Soccer Players. Int. J. Sport Nutr. Exerc. Metab..

[53] Tsuchiya Y., Yanagimoto K., Ueda H., Ochi E. (2019). Supplementation of eicosapentaenoic acid-rich fish oil attenuates muscle stiffness after eccentric contractions of human elbow flexors. J. Int. Soc. Sports Nutr..

[54] Barenie M.J., Freemas J.A., Baranauskas M.N., Goss C.S., Freeman K.L., Chen X., Dickinson S.L., Fly A.D., Kawata K., Chapman R.F. (2022). Effectiveness of a combined New Zealand green-lipped mussel and Antarctic krill oil supplement on markers of exercise-induced muscle damage and inflammation in untrained men. J. Diet. Suppl..

[55] Buonocore D., Verri M., Giolitto A., Doria E., Ghitti M., Dossena M. (2020). Effect of 8-week n-3 fatty-acid supplementation on oxidative stress and inflammation in middle- and long-distance running athletes: A pilot study. J. Int. Soc. Sports Nutr..

[56] Morishima T., Tsuchiya Y., Ueda H., Ochi E. (2020). Muscular endurance and muscle metabolic responses to 8 weeks of omega-3 polyunsaturated fatty acids supplementation. Physiol. Rep..

[57] Ramos-Campo D.J., Ávila-Gandía V., López-Román F.J., Miñarro J., Contreras C., Soto-Méndez F., Pedrol J.C.D., Luque-Rubia A.J. (2020). Supplementation of Re-Esterified Docosahexaenoic and Eicosapentaenoic Acids Reduce Inflammatory and Muscle Damage Markers after Exercise in Endurance Athletes: A Randomized, Controlled Crossover Trial. Nutrients.

[58] VanDusseldorp T.A., Escobar K.A., Johnson K.E., Stratton M.T., Moriarty T., Kerksick C.M., Mangine G.T., Holmes A.J., Lee M., Endito M.R. (2020). Impact of Varying Dosages of Fish Oil on Recovery and Soreness Following Eccentric Exercise. Nutrients.

[59] Kyriakidou Y., Wood C., Ferrier C., Dolci A., Elliott B. (2021). The effect of Omega-3 polyunsaturated fatty acid supplementation on exercise-induced muscle damage. J. Int. Soc. Sports Nutr..

[60] Loss L.C., Benini D., de Lima-E-Silva F.X., Möller G.B., Friedrich L.R., Meyer E., Baroni B.M., Schneider C.D. (2021). Effects of omega-3 supplementation on muscle damage after resistance exercise in young women: A randomized placebo-controlled trial. Nutr. Health.

[61] Tsuchiya Y., Ueda H., Yanagimoto K., Kato A., Ochi E. (2021). 4-week eicosapentaenoic acid-rich fish oil supplementation partially protects muscular damage following eccentric contractions. J. Int. Soc. Sports Nutr..

[62] Visconti L.M., Cotter J.A., Schick E.E., Daniels N., Viray F.E., Purcell C.A., Brotman C.B., Ruhman K.E., Escobar K.A. (2021). Impact of varying doses of omega-3 supplementation on muscle damage and recovery after eccentric resistance exercise. Metab. Open.

[63] Ayubi N., Purwanto B., Rejeki P.S., Kusnanik N.W., Herawati L., Komaini A., Mutohir T.C., Nurhasan N., Al Ardha M.A., Firmansyah A. (2022). Effect of acute omega 3 supplementation reduces serum tumor necrosis factor-alpha (TNF-a) levels, pain intensity, and maintains muscle strength after high-intensity weight training. Retos.

[64] Asjodi F., Rasekhi H., Mousavi S.E., Iravani O.M., Khazaei Y. (2023). The Combined Effect of Taurine and Omega-3 Supplementation on Delayed Onset Muscle Soreness in High-Intensity Eccentric Exercise. J. Iran. Med. Counc..

[65] Barquilha G., Dos Santos C.M.M., Caçula K.G., Santos V.C., Polotow T.G., Vasconcellos C.V., Gomes-Santos J.A.F., Rodrigues L.E., Lambertucci R.H., Serdan T.D.A. (2023). Fish Oil Supplementation Improves the Repeated-Bout Effect and Redox Balance in 20–30-Year-Old Men Submitted to Strength Training. Nutrients.

[66] Mackay J., Bowles E., Macgregor L.J., Prokopidis K., Campbell C., Barber E., Galloway S.D.R., Witard O.C. (2023). Fish oil supplementation fails to modulate indices of muscle damage and muscle repair during acute recovery from eccentric exercise in trained young males. Eur. J. Sport Sci..

[67] Yang S., He Q., Shi L., Wu Y. (2022). Impact of Antarctic krill oil supplementation on skeletal muscle injury recovery after resistance exercise. Eur. J. Nutr..

[68] Heileson J.L., Harris D.R., Tomek S., Ritz P.P., Rockwell M.S., Barringer N.D., Forsse J.S., Funderburk L.K. (2023). Long-Chain Omega-3 Fatty Acid Supplementation and Exercise-Induced Muscle Damage: EPA or DHA?. Med. Sci. Sports Exerc..

[69] Posnakidis G., Giannaki C.D., Mougios V., Pantzaris M., Patrikios I., Calder P.C., Sari D.K., Bogdanis G.C., Aphamis G. (2024). Effects of Supplementation with Omega-3 and Omega-6 Polyunsaturated Fatty Acids and Antioxidant Vitamins, Combined with High-Intensity Functional Training, on Exercise Performance and Body Composition: A Randomized, Double-Blind, Placebo-Controlled Trial. Nutrients.

[70] Makaje N., Ruangthai R., Sae-Tan S. (2024). Effects of Omega-3 Supplementation on the Delayed Onset Muscle Soreness after Cycling High Intensity Interval Training in Overweight or Obese Males. J. Sports Sci. Med..

[71] Rohatgi A. (2017). WebPlotDigitizer.

[72] Higgins J.P., Chandler J., Cumpston M., Li T., Page M.J., Welch V.A. (2023). Choosing effect measures and computing estimates of effect. Cochrane Handbook for Systematic Reviews of Interventions.

[73] Cohen J. (1992). A power primer. Psychol. Bull..

[74] Higgins J.P.T., Thompson S.G. (2002). Quantifying heterogeneity in a meta-analysis. Stat. Med..

[75] Anthony R., Macartney M.J., Peoples G.E. (2021). The Influence of Long-Chain Omega-3 Fatty Acids on Eccentric Exercise-Induced Delayed Muscle Soreness: Reported Outcomes Are Compromised by Study Design Issues. Int. J. Sport Nutr. Exerc. Metab..

[76] Anthony R., Macartney M.J., Heileson J.L., McLennan P.L., Peoples G.E. (2023). A review and evaluation of study design considerations for omega-3 fatty acid supplementation trials in physically trained participants. Nutr. Res. Rev..

[77] López-Seoane J., Martinez-Ferran M., Romero-Morales C., Pareja-Galeano H. (2021). N-3 PUFA as an ergogenic supplement modulating muscle hypertrophy and strength: A systematic review. Crit. Rev. Food Sci. Nutr..

[78] Harris W.S., Von Schacky C. (2004). The Omega-3 Index: A new risk factor for death from coronary heart disease?. Prev. Med..

[79] Paulsen G., Mikkelsen U.R., Raastad T., Peake J.M. (2012). Leucocytes, cytokines and satellite cells: What role do they play in muscle damage and regeneration following eccentric exercise?. Exerc. Immunol. Rev..

[80] Miotto P.M., Mcglory C., Bahniwal R., Kamal M., Phillips S.M., Holloway G.P. (2019). Supplementation with dietary ω-3 mitigates immobilization-induced reductions in skeletal muscle mitochondrial respiration in young women. FASEB J..

[81] Calder P.C. (2010). Omega-3 Fatty Acids and Inflammatory Processes. Nutrients.

[82] Peoples G.E., McLennan P.L. (2010). Dietary fish oil reduces skeletal muscle oxygen consumption, provides fatigue resistance and improves contractile recovery in the rat in vivo hindlimb. Br. J. Nutr..

[83] Slee E.L., McLennan P.L., Owen A.J., Theiss M.L. (2010). Low dietary fish-oil threshold for myocardial membrane n-3 PUFA enrichment independent of n-6 PUFA intake in rats. J. Lipid Res..

[84] Macartney M., Peoples G., Treweek T., McLennan P. (2019). Docosahexaenoic acid varies in rat skeletal muscle membranes according to fibre type and provision of dietary fish oil. Prostaglandins Leukot. Essent. Fat. Acids.

[85] Hawker G.A., Mian S., Kendzerska T., French M. (2011). Measures of adult pain: Visual Analog Scale for Pain (VAS Pain), Numeric Rating Scale for Pain (NRS Pain), McGill Pain Questionnaire (MPQ), Short-Form McGill Pain Questionnaire (SF-MPQ), Chronic Pain Grade Scale (CPGS), Short Form-36 Bodily Pain Scale (SF-36 BPS), and Measure of Intermittent and Constant Osteoarthritis Pain (ICOAP). Arthr. Care Res..

[86] Brancaccio P., Maffulli N., Limongelli F.M. (2007). Creatine kinase monitoring in sport medicine. Br. Med. Bull..

[87] Nikolaidis M.G., Kyparos A., Spanou C., Paschalis V., Theodorou A.A., Vrabas I.S. (2012). Redox biology of exercise: An integrative and comparative consideration of some overlooked issues. J. Exp. Biol..

