# Supplementary material for: Effects of LC n-3 PUFA Supplementation on Muscle Pain, Function, and Damage Markers in Healthy Young to Middle-Aged Adults Following Acute or Chronic Exercise: A Systematic Review and Meta-Analysis of Randomized Controlled Trials
Source: Nutrients. 2026 Apr 30;18(9):1447. doi: 10.3390/nu18091447 (PMC13165459; doi:10.3390/nu18091447)
Supplement: Supplementary file 1 [file nutrients-18-01447-s001.zip › nutrients-3762921-supplementary.pdf]

Supplementary Materials:

	Radom sequence generation (Selection bias)	Allocation concealment (Selection bias)	Blinding of participants and personnel (Performance bias)	Blinding of outcome assessment (detection bias)	Incomplete outcome data (attrition Bias)	Selective reporting (reporting bias)	Other biases
Toft et al (2000)	?	–	–	–	+	+	?
Lenn et al (2002)	?	–	?	?	–	+	?
Philips et al (2003)	?	–	+	?	–	+	?
Bloomer et al (2009)	?	–	+	?	+	+	+
Nieman et al (2009)	?	–	+	?	?	+	+
Poprzecki et al (2009	?	–	–	–	–	+	+
Tartibian et al (2009)	?	–	+	?	?	+	+
Jouris et al (2011)	–	–	–	–	–	+	+
Tartibian et al (2011)	?	–	+	?	?	+	+
Houghton & Onambele (2012)	?	–	+	?	+	–	–
Atashak et al (2013)	?	–	+	?	–	+	+
Rajabi et al (2013)	?	–	+	?	–	+	+
Dilorenzo et al (2014)	–	–	–	–	+	+	+
Gray et al (2014)	?	–	+	?	–	+	–
Lembke et al (2014)	?	–	–	–	+	+	+
Marques et al (2015)	–	–	–	–	+	+	+
Mickleborough et al (2015)	?	+	+	?	?	+	+

Corder et al (2016)	?	-	+	?	+	+	+
Tinsley et al (2016)	+	+	+	?	-	-	+
Tsuchiya et al (2016)	?	+	+	?	+	+	+
Jakeman et al (2017)	?	+	+	+	-	+	-
McKinley-Barnard et al (2017)	?	-	+	?	-	+	+
Ochi et al (2017)	+	+	+	?	+	+	+
Black et al (2018)	?	-	+	?	-	+	+
Philpott et al (2018)	?	-	+	?	+	+	-
Tsuchiya et al (2019)	+	+	+	?	-	+	+
Barenie et al (2020)	+	+	+	?	+	+	+
Buonocore et al (2020)	-	-	-	-	+	+	+
Morishima et al (2020)	+	+	+	?	-	+	+
Ramos-Campo et al (2020)	+	+	+	?	+	+	+
VanDusseldorp et al (2020)	?	-	+	?	+	+	+
Kyriakidou et al (2021)	+	-	-	-	+	+	+
Loss et al (2021)	+	+	+	+	+	+	+
Tsuchiya et al (2021)	+	+	+	?	-	+	+
Visconti et al (2021)	?	-	+	?	+	+	+
Ayubi et al (2022)	?	-	-	-	-	+	-
Asjodi et al (2023)	?	-	-	-	+	+	-
Barquilha et al (2023)	?	-	-	-	+	+	-
Mackay et al (2023)	?	-	+	?	-	+	+
Yang et al (2023)	+	-	+	?	-	+	-
Heileson et al (2024)	?	-	+	?	+	+	+
Posnakidis et al (2024)	+	-	+	?	+	+	-
Makaje et al (2024)	?	?	+	?	?	+	+

Figure S1. Risk of bias: authors' judgments regarding each risk of bias item for included studies using Cochrane Risk of Bias tool for randomized trials. **Abbreviations:** "+," "?" "−" within circles denote low-risk, unclear-risk, and high-risk, respectively.

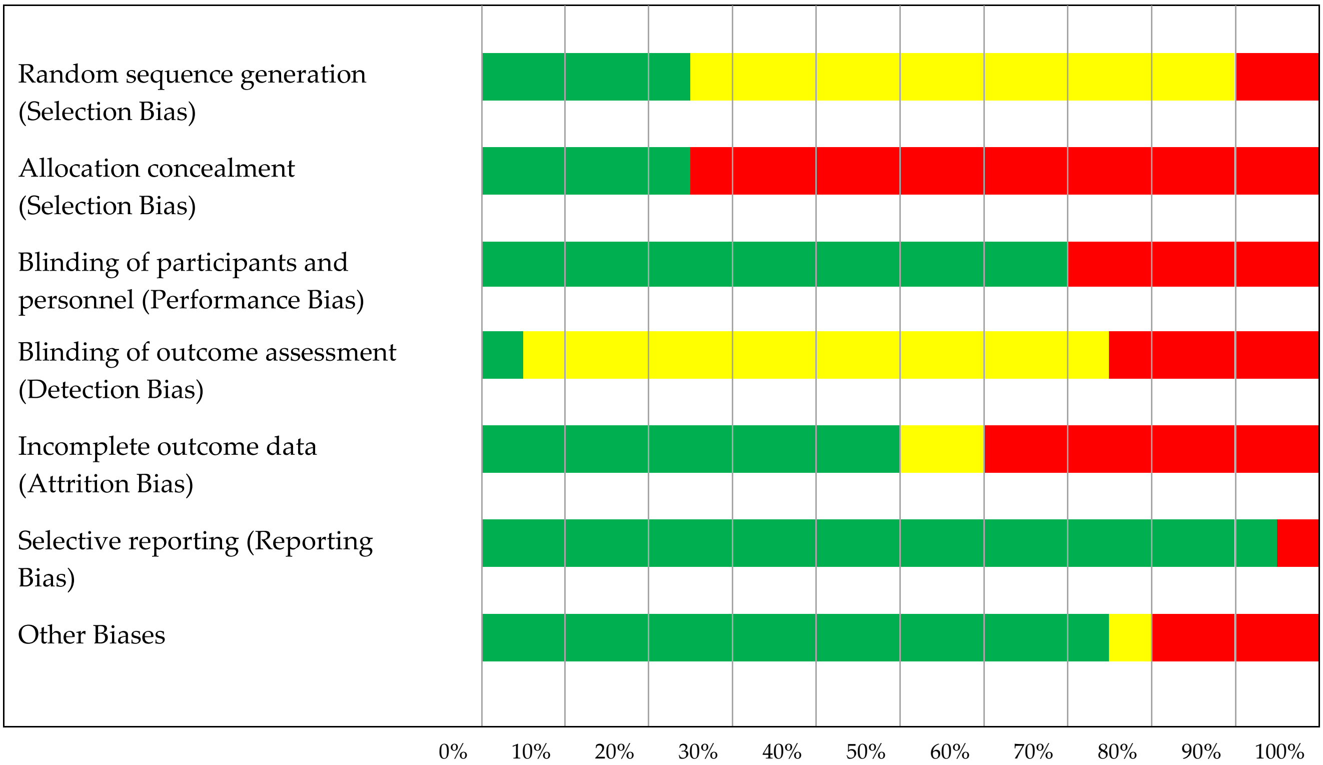

Figure S2: The most common problems found in the risk of bias in selected studies. **Abbreviations** Low-Risk Unclear-Risk High-Risk.

Methodological quality assessment for included studies: McMaster Critical Review Form for Quantitative Studies

**Study**	**Items**																**Total**	**%**
	**1**	**2**	**3**	**4**	**5**	**6**	**7**	**8**	**9**	**10**	**11**	**12**	**13**	**14**	**15**	**16**		
Toft et al (2000)	1	1	1	0	1	0	1	1	1	1	1	1	1	1	0	0	12	75
Lenn et al (2002)	1	1	1	0	1	1	1	1	1	1	1	1	0	1	0	0	12	75
Philips et al (2003)	1	1	1	1	1	1	1	1	1	1	1	1	1	1	1	1	16	100
Bloomer et al (2009)	1	1	1	1	1	0	1	1	1	1	1	1	1	1	1	1	15	93.8
Nieman et al (2009)	1	1	1	1	1	0	1	1	0	1	1	1	1	1	1	0	13	81.3
Poprzecki et al (2009)	1	1	0	0	1	0	1	1	1	1	1	1	1	1	1	0	12	75
Tartibian et al (2009)	1	1	1	1	1	1	1	1	1	1	1	1	1	1	1	1	16	100
Jouris et al (2011)	1	1	1	0	1	1	1	1	1	1	1	1	1	1	1	1	15	93.8
Tartibian et al (2011)	1	1	1	1	1	0	1	1	1	1	1	1	1	0	1	0	13	81.3
Houghton & Onambele (2012)	1	1	1	1	1	1	1	1	1	1	1	1	1	1	0	0	14	87.5
Atashak et al (2013)	1	1	1	1	1	1	1	1	1	1	1	1	1	1	1	0	15	93.8
Rajabi et al (2013)	1	1	1	1	1	0	0	1	1	1	0	1	0	1	0	0	10	62.5
Dilorenzo et al (2014)	1	1	1	0	1	1	1	1	1	1	1	1	1	1	1	0	14	87.5
Gray et al (2014)	1	1	1	1	1	1	1	1	1	1	1	1	1	1	1	1	16	100
Lembke et al (2014)	1	1	0	0	1	0	1	1	1	1	0	1	1	1	1	1	12	75
Marques et al (2015)	1	1	0	0	1	0	1	1	1	1	1	1	1	1	1	1	13	81.3
Mickleborough et al (2015)	1	1	1	1	1	1	1	1	1	1	1	1	1	1	1	1	16	100
Corder et al (2016)	1	1	1	1	1	1	1	1	1	1	1	1	1	1	1	0	15	93.8

Tinsley et al (2016)	1	1	1	1	1	1	1	1	1	1	1	1	0	1	0	1	14	87.5
Tsuchiya et al (2016)	1	1	1	1	1	1	1	1	1	1	1	1	0	1	1	1	15	93.8
Jakeman et al (2017)	1	1	1	1	1	1	1	1	1	1	1	1	1	1	1	0	15	93.8
McKinley-Barnard et al (2017)	1	1	1	1	1	1	1	1	1	1	1	1	1	1	1	0	15	93.8
Ochi et al (2017)	1	1	1	1	1	1	1	1	1	1	1	1	0	1	1	1	15	93.8
Black et al (2018)	1	1	1	1	1	1	1	1	1	1	1	1	1	1	1	0	15	93.8
Philpott et al (2018)	1	1	1	1	1	1	1	1	1	1	1	1	0	1	0	0	13	81.3
Tsuchiya et al (2019)	1	1	1	1	1	1	1	1	1	1	1	1	0	1	0	1	14	87.5
Barenie et al (2020)	1	1	1	1	1	1	1	1	1	1	1	1	0	1	0	1	14	87.5
Buonocore et al (2020)	1	1	0	0	1	1	1	1	1	1	1	1	1	1	0	0	12	75
Morishima et al (2020)	1	1	1	1	1	0	1	1	1	1	1	1	0	1	0	1	13	81.3
Ramos-Campo et al (2020)	1	1	1	1	1	1	1	1	1	1	1	1	1	1	0	1	15	93.8
VanDusseldorp et al (2020)	1	1	1	1	1	1	1	1	1	1	1	1	0	1	0	1	14	87.5
Kyriakidou et al (2021)	1	1	1	0	1	1	1	1	1	1	1	1	0	1	0	1	13	81.3
Loss et al (2021)	1	1	1	1	1	1	1	1	1	1	1	1	0	1	0	1	14	87.5
Tsuchiya et al (2021)	1	1	1	1	1	1	1	1	1	1	1	1	1	1	0	0	14	87.5
Visconti et al (2021)	1	1	1	1	1	1	1	1	1	1	1	1	1	1	0	1	15	93.8
Ayubi et al (2022)	1	1	1	0	1	1	1	1	1	1	0	1	0	1	0	0	11	68.75
Asjodi et al (2023)	1	1	1	0	1	1	1	1	1	1	1	1	0	1	0	0	12	75
Barquilha et al (2023)	1	1	0	0	1	0	1	1	1	1	0	1	1	1	1	1	12	75
Mackay et al (2023)	1	1	1	1	1	1	1	1	1	1	1	1	0	1	0	1	14	87.5
Yang et al (2023)	1	1	1	1	1	1	1	1	1	1	1	1	0	1	1	1	15	93.8
Heileson et al (2024)	1	1	1	1	1	1	1	1	1	1	0	1	1	1	1	1	15	93.8
Posnakidis et al (2024)	1	1	1	1	1	1	1	1	1	1	1	1	0	1	0	1	14	87.5
Makaje et al (2024)	1	1	1	1	1	1	1	1	1	1	1	1	0	1	0	1	14	87.5

Figure S3. Methodological quality assessment for included studies: McMaster Critical Review Form for Quantitative Studies. **Abbreviations:** 1 = fulfilled criterion; 0 = not fulfilled criterion; Item 1, study purpose; item 2, literature review; item 3, study design; item 4, blinding; item 5, sample description; item 6, sample size; item 7, ethics and consent; item 8, validity of outcomes; item 9, reliability of outcomes; item 10, intervention description; item 11, statistical significance; item 12, statistical analysis; item 13, clinical importance; item 14, conclusions; item 15, clinical implications; item 16, study limitations. Methodological quality according to the PEDro Scale

**Study**	**Items**											**Total**	**%**
	**1**	**2**	**3**	**4**	**5**	**6**	**7**	**8**	**9**	**10**	**11**		
Toft et al (2000)	1	1	0	1	0	0	0	1	0	1	1	5	50
Lenn et al (2002)	1	1	0	1	0	0	0	0	0	1	1	4	40
Philips et al (2003)	1	1	0	1	1	1	0	1	1	1	1	8	80
Bloomer et al (2009)	1	1	1	1	1	1	0	0	0	1	1	7	70
Nieman et al (2009)	1	1	0	1	1	1	0	1	1	1	1	8	80
Poprzecki et al (2009)	1	1	0	1	0	0	0	1	1	1	1	6	60
Tartibian et al (2009)	1	1	0	1	1	1	0	1	1	1	1	8	80
Jouris et al (2011)	1	0	0	0	0	0	0	1	1	1	1	4	40
Tartibian et al (2011)	1	1	0	1	1	1	0	1	0	1	1	7	70
Houghton & Onambele (2012)	1	1	0	0	1	1	0	1	0	1	1	6	60
Atashak et al 2013)	1	1	0	1	1	1	0	1	1	1	1	8	80
Rajabi et al (2013)	1	1	0	0	1	1	0	1	0	1	1	6	60

Dilorenzo et al (2014)	1	1	0	1	0	0	0	1	0	1	1	5	50
Gray et al (2014)	1	1	0	1	1	1	0	1	1	1	1	8	80
Lembke et al (2014)	1	1	0	0	0	0	0	1	0	1	1	4	40
Marques et al (2015)	1	0	0	0	0	0	0	1	1	1	1	4	40
Mickleborough et al (2015)	1	1	0	1	1	1	0	1	0	1	1	7	70
Corder et al (2016)	1	1	0	1	1	1	0	0	0	1	1	6	60
Tinsley et al (2016)	1	1	1	1	1	1	0	1	0	1	1	8	80
Tsuchiya et al (2016)	1	1	1	0	1	1	0	1	0	1	1	7	70
Jakeman et al (2017)	1	1	1	0	1	1	1	1	0	1	1	8	80
McKinley-Barnard et al (2017)	1	1	0	1	1	1	0	1	0	1	1	7	70
Ochi et al (2017)	1	1	1	1	1	1	0	1	0	1	1	8	80
Black et al (2018)	1	1	0	0	1	1	0	0	0	1	1	5	50
Philpott et al (2018)	1	0	0	1	1	1	0	1	0	1	1	6	60
Tsuchiya et al (2019)	1	1	1	1	1	1	0	1	0	1	1	8	80
Barenie et al (2020)	1	1	0	1	1	1	0	1	0	1	1	7	70
Buonocore et al (2020)	1	0	0	0	0	0	0	1	0	1	1	3	30
Morishima et al (2020)	1	1	1	1	1	1	0	1	0	1	1	8	80
Ramos-Campo et al (2020)	1	1	1	1	1	1	0	0	0	1	1	7	70
VanDusseldorp et al (2020)	1	1	0	1	1	1	0	0	0	1	1	6	60
Kyriakidou et al (2021)	1	1	0	1	1	0	0	0	0	1	1	5	50
Loss et al (2021)	1	1	1	1	1	1	1	1	1	1	1	10	100
Tsuchiya et al (2021)	1	1	1	1	1	1	0	1	0	1	1	8	80
Visconti et al (2021)	1	1	1	1	1	0	1	1	1	1	1	9	90
Ayubi et al (2022)	1	1	0	1	1	0	0	1	0	1	1	6	60
Asjodi et al (2023)	1	1	0	1	0	0	0	1	1	1	1	6	60
Barquilha et al (2023)	1	1	0	0	0	0	0	0	0	1	1	3	30
Mackay et al (2023)	1	1	0	1	1	1	0	1	0	1	1	7	70
Yang et al (2023)	1	1	0	1	1	1	0	1	0	1	1	7	70
Heileson et al (2024)	1	1	0	1	1	1	0	1	0	1	1	7	70
Posnakidis et al (2024)	1	1	0	1	1	1	0	1	1	1	1	8	80
Makaje et al (2024)	1	1	0	1	1	1	0	1	1	1	1	8	80

Figure S4. Methodological quality according to the PEDro Scale. **Abbreviation:** 1 = Criterion met; 0 = Criterion not met, Item 1 = eligibility criteria were specified, 2 = whether participants were randomly allocated to groups, 3 = whether allocation was concealed, 4 = the groups were similar at baseline regarding the most important prognostic indicators, 5 =blinding of subjects,6 = therapists administering therapy were blinded, 7 = assessors were blinded, 8 = measures of at least one key outcomes were obtained from more than 85% of the subjects initially allocated to groups, 9 = all subjects for whom outcome measures were available received the treatment or control condition as allocated or, where this was not the case, data for at least one key outcome was analyzed by "intention to treat", 10 = the results of between-group statistical comparisons are reported for at least one key outcome, 11 = the study provides both point measures and measures of variability for at least one key outcome.

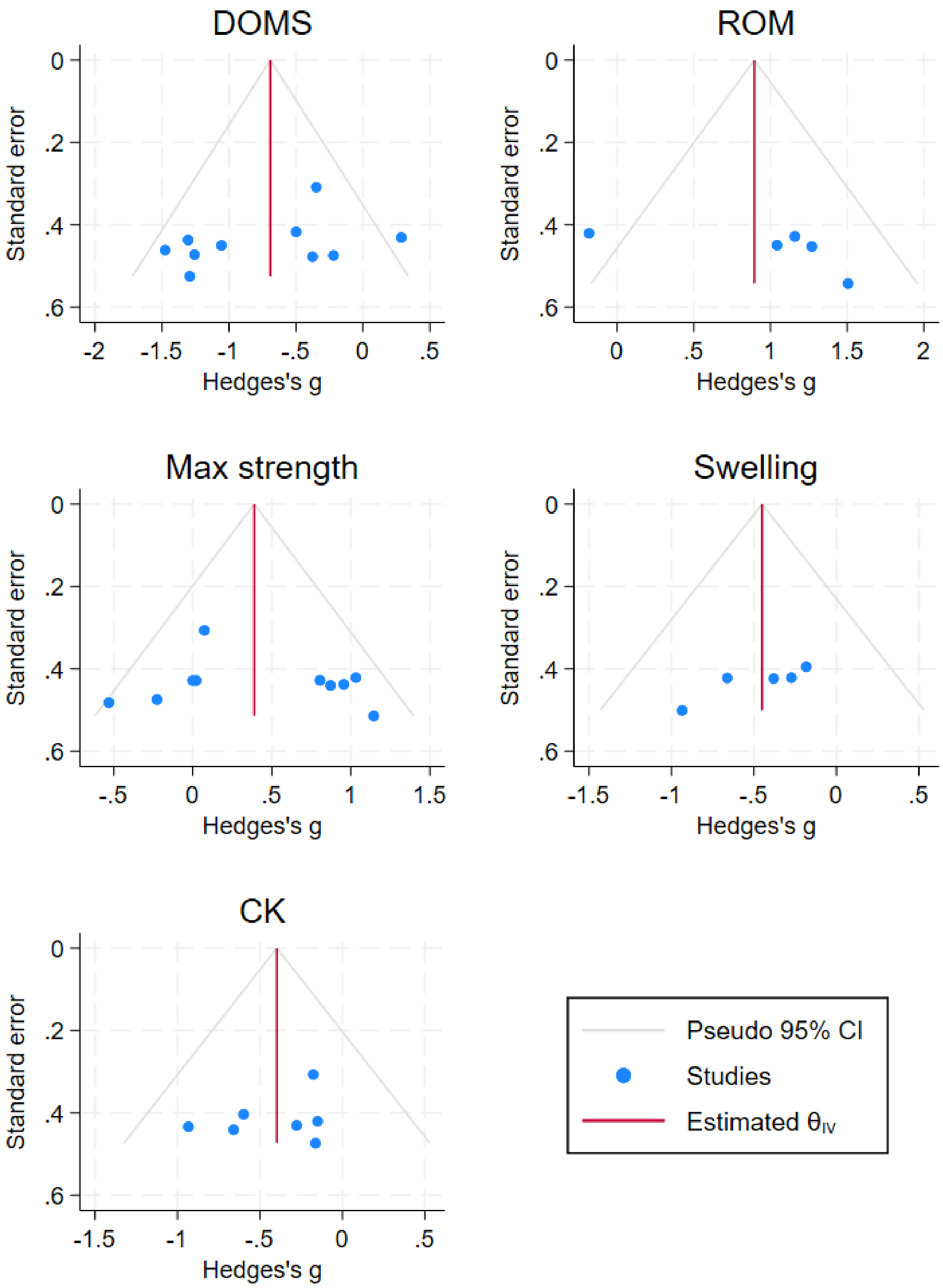

Figure S5. Funnel plots of delayed onset muscle soreness (DOMS), creatine kinase (CK), muscle strength, range of motion (ROM), and muscle swelling.

Supplementary Tables for Leave-One-Out Meta-Analysis:

Table S1. Leave-one-out meta-analysis (DOMS outcome) [a]

Study excluded	Hedge's g (95% CI)	p-value
Dilorenzo et al (2014)	-0.82 (-1.26, -0.38)	<0.0005
Gray (2014)	-0.86 (-1.22, -0.50)	<0.0005

Heileson et al (2024)	-0.66 (-1.06, -0.27)	0.001
Mackay et al (2023) (hamstring)	-0.78 (-1.17, -0.38)	<0.0005
Mackay et al (2023) (quads)	-0.76 (-1.16, -0.36)	<0.0005
Ochi et al (2017)	-0.72 (-1.15, -0.29)	0.001
Philpott et al (2018)	-0.70 (-1.11, -0.28)	0.001
Tsuchiya et al (2016)	-0.68 (-1.10, -0.27)	0.001
Tsuchiya et al (2019)	-0.70 (-1.12, -0.29)	0.001
Tsuchiya et al (2021)	-0.79 (-1.23, -0.35)	<0.0005
All studies included	-0.75 (-1.14, -0.36)	<0.0005

[a] Random-effects multilevel REML meta-analysis estimates, excluding a single study

Table S2. Leave-one-out meta-analysis (CK outcome) [a]

Study excluded	Hedge's g (95% CI)	p-value
Dilorenzo et al (2014)	-0.47 (-0.82, -0.12)	0.008
Gray (2014)	-0.41 (-0.74, -0.09)	0.011
Heileson et al (2024)	-0.43 (-0.76, -0.11)	0.008
Mackay et al (2023)	-0.42 (-0.74, -0.11)	0.009
Philpott et al (2018)	-0.36 (-0.68, -0.04)	0.027
Tsuchiya et al (2016)	-0.36 (-0.69, -0.04)	0.028
Tsuchiya et al (2021)	-0.32 (-0.64, 0.00)	0.050
All studies included	-0.40 (-0.70, -0.10)	0.009

[a] Random-effects REML meta-analysis estimates, excluding a single study

Table S3. Leave-one-out meta-analysis (maximum strength outcome) [a]

Study excluded	Hedge's g (95% CI)	p-value
Dilorenzo et al (2014)	0.52 (0.09, 0.94)	0.017
Gray (2014)	0.51 (0.09, 0.92)	0.016
Heileson et al (2024)	0.39 (-0.01, 0.79)	0.056
Mackay et al (2023) (hamstring)	0.49 (0.15, 0.83)	0.005
Mackay et al (2023) (quads)	0.47 (0.10, 0.84)	0.014
Ochi et al (2017)	0.40 (0.00, 0.81)	0.053
Philpott et al (2018)	0.51 (0.09, 0.92)	0.017
Tsuchiya et al (2016)	0.38 (-0.02, 0.77)	0.060
Tsuchiya et al (2019)	0.38 (-0.01, 0.77)	0.053
Tsuchiya et al (2021)	0.41 (0.00, 0.83)	0.051
All studies included	0.45 (0.07, 0.83)	0.020

[a] Random-effects multilevel REML meta-analysis estimates, excluding a single study

Table S4. Leave-one-out meta-analysis (ROM outcome) [a]

Study excluded	Hedge's g (95% CI)	p-value
Heileson et al (2024)	1.22 (0.77, 1.67)	<0.0005
Ochi et al (2017)	0.91 (0.14, 1.67)	0.020
Tsuchiya et al (2016)	0.87 (0.12, 1.63)	0.023
Tsuchiya et al (2019)	0.81 (0.14, 1.49)	0.019
Tsuchiya et al (2021)	0.85 (0.12, 1.58)	0.023
All studies included	0.93 (0.33, 1.53)	0.002

[a] Random-effects REML meta-analysis estimates, excluding a single study

Table S5. Leave-one-out meta-analysis (swelling outcome) [a]

**Study excluded**	**Hedge's g (95% CI)**	**p-value**
Heileson et al (2024)	-0.50 (-0.92, -0.08)	0.021
Ochi et al (2017)	-0.47 (-0.89, -0.05)	0.029
Tsuchiya et al (2016)	-0.53 (-0.96, -0.10)	0.015
Tsuchiya et al (2019)	-0.37 (-0.77, 0.04)	0.076
Tsuchiya et al (2021)	-0.40 (-0.82, 0.03)	0.066
All studies included	-0.45 (-0.83, -0.07)	0.019

[a] Random-effects REML meta-analysis estimates, excluding a single study
